# HAPLN1 knockdown inhibits heart failure development via activating the PKA signaling pathway

**DOI:** 10.1186/s12872-024-03861-8

**Published:** 2024-04-05

**Authors:** Tao Yan, Shushuai Song, Wendong Sun, Yiping Ge

**Affiliations:** 1 Department of Cardiology, Zibo Municipal Hospital, Ward 1, No. 139 Huangong Road, Linzi District, Zibo City, Shandong Province 255400 China; 2 Department of Cardiology, Qingdao Fuwai Cardiovascular Hospital, No. 201 Nanjing Road, Shibei District, Qingdao City, Shandong Province 266034 China; 3 Department of Cardiology, Zibo Municipal Hospital, No. 139 Huangong Road, Linzi District, Zibo City, Shandong Province 255400 China

**Keywords:** Heart failure, HAPLN1, PKA signaling pathway

## Abstract

**Background:**

Heart failure (HF) is a heterogeneous syndrome that affects millions worldwide, resulting in substantial health and economic burdens. However, the molecular mechanism of HF pathogenesis remains unclear.

**Methods:**

HF-related key genes were screened by a bioinformatics approach.The impacts of HAPLN1 knockdown on Angiotensin II (Ang II)-induced AC16 cells were assessed through a series of cell function experiments. Enzyme-linked immunosorbent assay (ELISA) was used to measure levels of oxidative stress and apoptosis-related factors. The HF rat model was induced by subcutaneous injection isoprenaline and histopathologic changes in the cardiac tissue were assessed by hematoxylin and eosin (HE) staining and echocardiographic index. Downstream pathways regulated by HAPLN1 was predicted through bioinformatics and then confirmed in vivo and in vitro by western blot.

**Results:**

Six hub genes were screened, of which HAPLN1, FMOD, NPPB, NPPA, and COMP were overexpressed, whereas NPPC was downregulated in HF. Further research found that silencing HAPLN1 promoted cell viability and reduced apoptosis in Ang II-induced AC16 cells. HAPLN1 knockdown promoted left ventricular ejection fraction (LVEF) and left ventricular fraction shortening (LVFS), while decreasing left ventricular end-systolic volume (LVESV) in the HF rat model. HAPLN1 knockdown promoted the levels of GSH and suppressed the levels of MDA, LDH, TNF-α, and IL-6. Mechanistically, silencing HAPLN1 activated the PKA pathway, which were confirmed both in vivo and in vitro.

**Conclusion:**

HAPLN1 knockdown inhibited the progression of HF by activating the PKA pathway, which may provide novel perspectives on the management of HF.

**Supplementary Information:**

The online version contains supplementary material available at 10.1186/s12872-024-03861-8.

## Introduction

Cardiovascular disease, a leading contributor to human mortality, encompasses a spectrum of conditions that ultimately lead to heart failure (HF) [1]. HF is a multifaceted clinical syndrome arising from impairment [2]. It is characterized by the heart’s inability to pump sufficient blood and oxygen to meet the metabolic needs of organs [3]. Approximately 64.3 million individuals globally are affected by HF, and the lifetime risk of HF varies from 20 to 45% in individuals aged over 45 years, depending on their race and ethnicity [4, 5]. Moreover, after hospitalization for cardiac decompensation, about 10% of patients die within 90 days of hospital discharge [6]. Therefore, it is important to further investigate the pathogenesis of HF and enhance treatment strategies.

The pathogenesis of HF mainly consists of myocardial remodeling, impaired contractility, neurohormonal activation, and inflammation and oxidative stress [7–9], which are regulated by a variety of genes. By modulating the lncRNA TUG1/miR-129-5p/ATG7 axis, ETS2 increases apoptosis and autophagy in HF [10]. H3K9me2 regulates BDNF expression via G9a, thereby contributing to the progression of HF [11]. Mutations in genes associated with myofibrillar proteins, such as MYH7, ACTC1, and TPM1, are linked to hypertrophic cardiomyopathy, impacting the heart’s ability to pump effectively [12, 13]. However, the molecular mechanisms governing the pathogenesis of HF remain incompletely understood due to their inherent complexity.

HAPLN1 (Hyaluronan and proteoglycan link protein 1), acknowledged as a cartilage link protein, was initially discovered in the proteoglycan fraction isolated from bovine articular cartilage [14]. Multiple studies have shown that HAPLN1 is involved in tumor progression, such as gastric cancer, pancreatic cancer, and myeloma [15–17]. However, the role of HAPLN1 in HF and the exact mechanisms have rarely been reported. Deletion of HAPLN1 induces atrial septal and myocardial defects in mice and reduces levels of multifunctional proteoglycans [18]. The HAPLN family, including HAPLN1, HAPLN2, HAPLN3, and HAPLN4, participates in the phospholipase-C pathway and integrin pathway, which have an important correlation with HF [19, 20]. These discoveries suggest that HAPLN1 may play a role in the development of HF. Moreover, it is worth noting that HAPLN1 maintains the aggregation and binding functionality of extracellular matrix (ECM) molecules, including hyaluronic acid and proteoglycan, solidifying the overall macromolecular structure of the ECM [21]. The ECM plays an important role in cardiac homeostasis by providing structural support, facilitating force transmission, and transmitting key signals to cardiomyocytes, vascular cells, and mesenchymal cells [22]. Consequently, investigating the role and mechanism of HAPLN1 in HF is of paramount significance. This study identified six key genes, namely FMOD, NPPB, NPPA, COMP, HAPLN1, and NPPC, through bioinformatics analysis of the GSE116250 and GSE135055 datasets. Receiver operating characteristic (ROC) curve analysis demonstrated that HAPLN1 could serve as diagnostic biomarkers for HF. Further investigations revealed that silencing HAPLN1 inhibited apoptosis, cardiomyocyte hypertrophy, and oxidative stress both in vivo and in vitro might through activation of the PKA signaling pathway. This study delves into the underlying pathogenesis of HF and identifies potential therapeutic targets for HF treatment.

## Materials and methods

### Datasets collection and processing

We used the keywords “heart failure” to search for microarray expression datasets related to HF in the Gene Expression Omnibus (GEO) database (http://www.ncbi.nlm.nih.gov/geo). After a thorough examination, the datasets GSE116250 and GSE135055 were chosen for analysis. In the GSE116250 dataset, 14 normal tissue samples and 50 HF samples were chosen to identify differentially expressed genes (DEGs). Similarly, in the GSE135055 dataset, nine normal tissue samples and 21 HF tissue samples were selected for gene differential expression analysis. The DEGs between HF and normal samples were identified using the GEO2R tool (http://www.ncbi.nlm.nih.gov/geo/geo2r) with a screening criterion of p-value ≤ 0.05 and |logFC| ≥ 2. Furthermore, the visualization of DEGs was performed using the pheatmap and ggplot2 packages in the R software (version 3.6.0).

### Gene set enrichment analysis (GSEA)

The GSEA (Gene set enrichment analysis) was performed using the ClusterProfiler package in the R software. The analysis included both the datasets, GSE116250 and GSE135055. For the analysis comparing HF and normal tissues, the gene sets “KEGG_CELL_CYCLE” and “KEGG_APOPTOSIS” were employed.

### Identification of overlapping DEGs and functional enrichment analysis

We used a Venn diagram analysis (https://bioinfogp.cnb.csic.es/tools/venny/) to identify overlapping DEGs between the GSE116250 and GSE135055 datasets. The overlapping DEGs were submitted to the Database for Annotation, Visualization and Integrated Discovery (DAVID; https://david.ncifcrf.gov/) online database for Gene Ontology (GO) and Kyoto Encyclopedia of Genes and Genomes (KEGG) enrichment analyses. The GO analysis includes biological processes (BP), cellular components (CC), and molecular functions (MF). Furthermore, the Sangerbox platform was utilized to visualize the results.

### Protein-protein interaction (PPI) network construction and hub gene identification

For the PPI network analysis, an interaction score threshold greater than 0.4 was applied in the Search Tool for the Retrieval of Interacting Genes/Proteins (STRING; https://string-db.org/) database. The PPI network was visualized using Cytoscape (version 3.7.1, http://www.cytoscape.org). For the identification of key genes, a total of 11 topological analysis methods, including, Degrre, Edge Percolated component (EPC), Maximum neighborhood component (MNC), Density of Maximum Neighborhood Component (DMNC), Maximal Clique Centrality (MCC) and six centralities (Botteleneck, EcCentricity, Closeness, Radiality, Betweenness, Stress), available in the cytoHubba plugin of the Cytoscape, were employed.

### Hub genes analysis

Utilizing data from the GSE116250 dataset, a series of analyses for hub genes were conducted, including GO, expression distribution, correlation, and principal component analysis (PCA). The GO enrichment chord chart for the chosen hub genes was generated by the circlize package in the R software. For visualizing gene expression ridgelines, the ggridges package was employed. Then, correlations between hub genes were explored using the corrplot and hmisc packages in the R software. The PCA was conducted with the factoextra package. To assess the diagnostic value of HAPLN1 for HF, ROC curve analysis was conducted using the pROC package based on the GSE116250 and GSE135055 datasets in the R software.

### Cell culture

The human cardiomyocyte cell line, AC16 cells, were acquired from Icellbioscience Biotechnology Co., Ltd. (Shanghai, China). The cells were grown in Dulbecco’s modified Eagle medium (Thermo Fisher Scientific, Massachusetts, USA) supplemented with 10% fetal bovine serum (Thermo Fisher Scientific) and 1% penicillin/streptomycin. Culture flasks were placed in a humidified environment with a gas mixture of 95% air and 5% CO_2_ at 37 °C.

### Construction of HF cell model and cell transfection

In this study, HF cell models were constructed utilizing AC16 cells with induction by Angiotensin II (Ang II) or doxorubicin (DOX). Specifically, 1 mmol/L of Ang II (Abcam, Cambridge, UK) was added, and the cells were grown for 48 h [23]. For DOX induction, AC16 cells were incubated in six-well plates with 2 µmol/L DOX (Sigma-Aldrich, 25316-40-9, Shanghai, China) for 24 h [24]. Short hairpin RNAs (shRNAs) targeting HAPLN1 and negative control (NC) were synthesized by Shanghai Integrated Biotech Solutions Co., Ltd. (Shanghai, China). Subsequently, the shRNAs were introduced into pcDNA3.1(+), and AC16 cells were seeded in a six-well plate containing 1 µg of pcDNA3.1(+)-HAPLN1 or an empty pcDNA3.1(+) vector. The transfections were performed using Lipofectamine® 3000 reagent (Thermo Fisher Scientific; L3000075) and lentivirus vector following the manufacturer’s guidelines for six hours at a temperature of 37 °C. The empty pcDNA3.1(+) vector was served as a negative control. The sh-NC and sh-HAPLN1 sequences used in this study are provided in Supplementary Table 1.

### RNA extraction and quantitative reverse transcription-polymerase chain reaction (RT-qPCR)

RT-qPCR was conducted to assess both the mitochondrial DNA (mtDNA) copy number and the levels of hub genes. Following the manufacturer’s guidelines, the extraction of total RNA was carried out using TRIzol reagent (Thermo Fisher Scientific; A33251), followed by the generation of cDNA from 1 µg of total RNA with a Prime-Script RT Reagent Kit (Thermo Fisher Scientific; 10,928,042). The RT-qPCR was performed on the 7500 real-time PCR System (Thermo Fisher Scientific) with the SYBR Premix Ex Taq (Takara, Dalian, China). Comparative expression of target genes was determined utilizing the 2^−∆∆CT^ method, where ∆CT equals target gene - GAPDH, and ∆∆ equals experiment – control. GAPDH was employed as the internal reference gene. The RT-qPCR primer sequences used in this study are shown in Supplementary Table 2.

### Cell counting kit-8 (CCK-8) assay

Cell viability was assessed using a CCK-8 kit (Solarbio, Beijing, China; CA1210) following the manufacturer’s instructions. AC16 cells were seeded into 96-well plates and allowed to reach 75% confluence. After transfection, a total of 10 µL CCK-8 reagent was added to each well and incubated for 1 h at 37 °C. A microplate reader (DALB, Shanghai, China) was used to measure the optical density (OD) at 450 nm.

### Flow cytometry

After 48 h of transfection, AC16 cells treated with Ang II were harvested by trypsinization. The cells were centrifuged at 1000 rpm for 4 min, washed with buffer three times, and suspended at a cell density of 3 × 10^6^ cells/mL. Apoptotic cells were then treated by adding Annexin V-FITC (50 µg/mL; Thermo Fisher Scientific; A23202) and propidium iodide (PI; 10 µg/mL; Thermo Fisher Scientific; BMS500PI) for 10 min at 37 °C. Subsequently, the cells that had been labeled were analyzed for apoptosis using a FACScan flow cytometer (Becton, Dickinson and Company, New Jersey, USA).

### Western blot assay

The cell lysate was obtained by employing RIPA lysis buffer (Solarbio; R1200) supplemented with a protease inhibitor cocktail set (Solarbio; A8260). The protein content was subsequently assessed using a bicinchoninic acid assay kit (Thermo Fisher Scientific; 23,227). To separate proteins, sodium dodecyl sulfate-polyacrylamide gel electrophoresis (SDS-PAGE) was employed. The proteins were then transferred from the gel to polyvinylidene fluoride (PVDF) membranes (Roche, Basel, Switzerland; 03010040001) using a current of 200 mA for 50 to 60 min, based on protein size. Following this, the membranes underwent incubation with primary antibodies, specifically anti-ANP (1:2000; Abcam; ab181242), anti-BNP (1:2000; Abcam; ab309127), anti-MMP-1 (1:2000; Abcam; ab52631), anti-cAMP (1:2000; Abcam; ab76238), anti-PKA (1:2000; Abcam; ab32514), anti-PKC (1:2000; Abcam; ab32376), anti-PLB (1:2000; Abcam; ab85146), anti-p-PLB (1:500; Abcam; ab62170), anti-CAMKII (1:2000; Abcam; ab134041), p-CaMKII (1:500; Thermo Fisher, PA5-37833), and anti-GAPDH (Abcam; ab8245) overnight at 4 °C. After two washes with TBST, the membranes were incubated with HRP-labeled secondary antibodies for 1 h at room temperature. Subsequently, immunoreactive bands were visualized using an ECL reagent (Amersham, Little Chalfont, UK; RPN2232).

### Enzyme-linked immunosorbent assay (ELISA)

The levels of the ATP, complex I and V, glutathione (GSH), malondialdehyde (MDA), and reactive oxygen species (ROS), lactate dehydrogenase (LDH), tumor necrosis factor-alpha (TNF-α), and interleukin-6 (IL-6) were quantified by the ELISA kits (Esebio Biotechnology Co., Ltd., Shanghai, China), following the manufacturers’ instructions.

### Exploration of the downstream mechanisms of HAPLN1

From the Genecards database (https://www.genecards.org/), a total of 15,852 genes associated with HF were sourced. Moreover, 500 genes co-expressed with HAPLN1 were acquired from the Coxpresdb database (https://coxpresdb.jp/). Then, by drawing a Venn diagram, overlapping genes were screened out. KEGG functional enrichment analysis was conducted on the shared genes to identify the pivotal pathways associated with HF.

### Construction of the HF animal model

The animal experimental procedures were in compliance with the Guide for the Care and Use of Laboratory Animals (US National Institutes of Health Publications, 8th edition, 2011). A total of 24 specific pathogen-free grade Sprague-Dawley (SD) male rats (180–200 g, aged 6–8 weeks; SPF Biotechnology Co., Ltd., Beijing, China) were housed individually in temperature-controlled rooms (22 °C) with adequate access to water and food. Rats were injected subcutaneously with 0.9% saline-dissolved isoproterenol (ISO) at a dose of 2.5 mg/kg once a day for 4 weeks to induce the HF model. Rats in the sham-operated group were injected with equal amounts of saline in the same manner. Adenoviral vectors encoding control shRNA or HAPLN1 shRNA (2 × 10^11^ plaque forming units/mL; Genomeditech Co. Shanghai, China) were injected intravenously into the tail of rats. The rats were randomly divided into 4 groups (6 rats in each group), including sham, HF, HF + sh-NC, and HF + sh-HAPLN1 groups. After 4 weeks, mice were euthanized with isoflurane inhalation and then serum and cardiac tissue were collected for subsequent experiments.

### Cardiac functions examination

Rats were anesthetized by inhalation of 2% isoflurane. Then the left ventricular function of rats, including left ventricular ejection fraction (LVEF), left ventricular fraction shortening (LVFS), and left ventricular end-systolic volume (LVESV) were measured using the VINNO 6VET ultrasound system (Vinno Technology, Suzhou, China). In addition, after the cardiac tissue was removed and washed in pre-cooled saline, the heart weight/body weight (HW/BW) ratio was calculated.

### Hematoxylin and eosin (HE) staining

After fixation in 4% paraformaldehyde and embedding in paraffin, cardiac tissues were sectioned to 4 μm thickness. Following deparaffinization with xylene, the sections underwent hydration in a series of decreasing ethanol concentrations (100–70%). Subsequently, they were washed in tap water and stained with hematoxylin and eosin. Following ethanol dehydration and xylene cleaning, the sections were sealed with neutral gum. The tissue morphology was observed under a microscope (Olympus, Tokyo, Japan).

### Statistical analysis

Experiments were conducted at least three times, and data analysis was performed using GraphPad Prism 7.0 software. The measured data were represented as the mean ± standard deviation (SD). Statistical differences between two groups were assessed using t-test. For multiple group comparisons, analysis of variance (ANOVA) was applied, followed by tukey’s multiple comparisons test. A significance level of *p* < 0.05 was used to determine statistical significance.

## Results

### Data processing

**Fig. 1:**
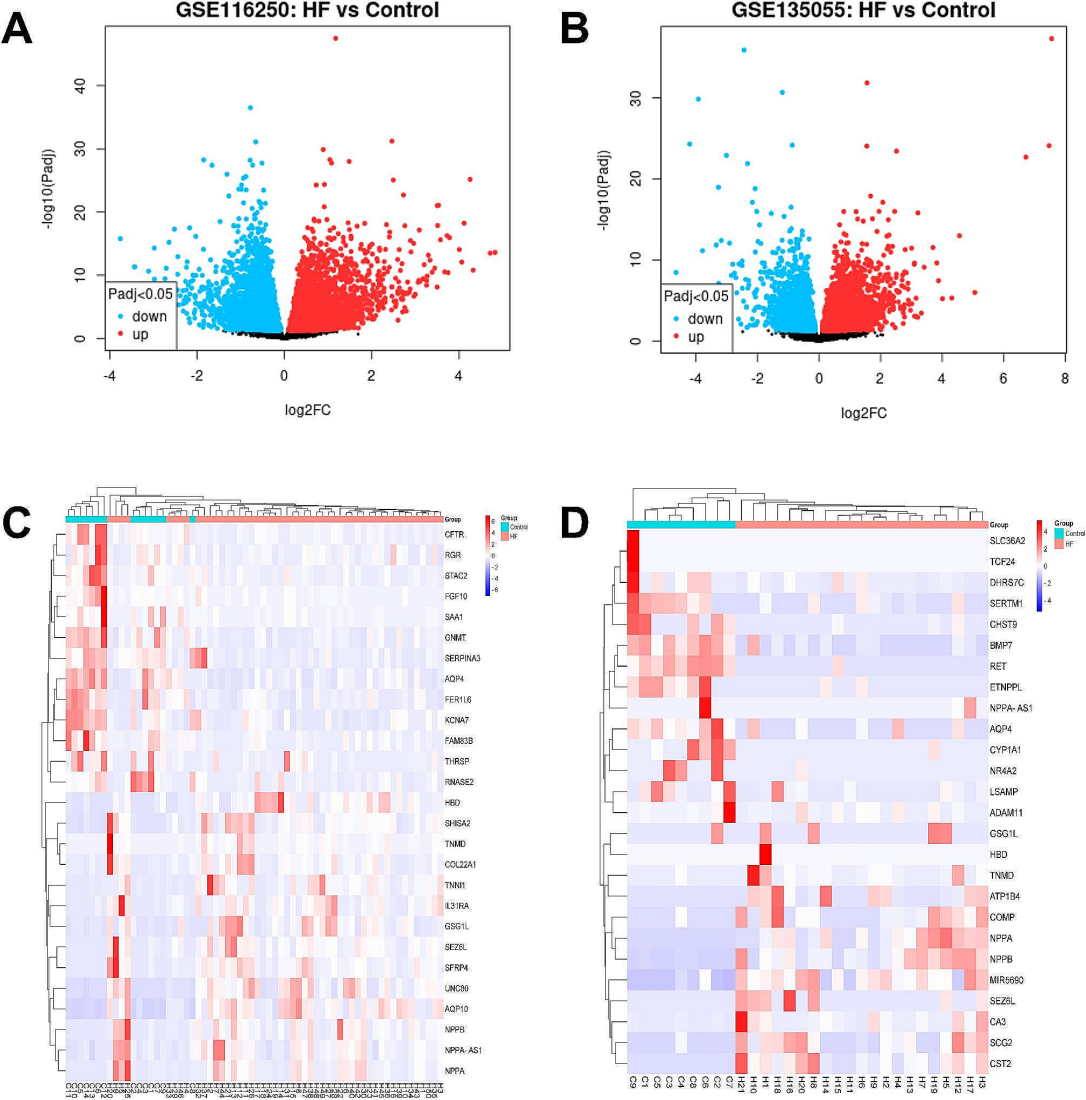
The distribution of DEGs be visualized using the volcano plots. (**A**) The volcano plot of GSE116250 dataset, including 182 DEGs (130 up-regulated genes and 52 down-regulated genes). (**B**) The volcano plot of GSE135055 dataset, comprising 171 DEGs (121 up-regulated genes and 50 down-regulated genes). The red dots represent the upregulated DEGs, the blue dots represent the downregulated DEGs, and the black dots represent genes with no significant differences. (**C**-**D**). The heatmap displayed the expression levels of the top 30 DEGs identified from the datasets GSE116250 and GSE135055, respectively. The change in color from red to blue indicates a progressive decrease in gene expression in the sample. Each bioinformatics analysis was performed independently three times. *Notes* DEGs, differently expressed genes

For our research, we selected two gene expression profiling datasets: GSE116250 and GSE135055. The read counts were normalized for each sample, and the results demonstrated a high level of consistency in the median values across all samples. This suggested that both the GSE116250 and GSE135055 datasets satisfied the predetermined criteria for further analysis (Supplementary Fig. 1). DEGs analysis was conducted on the two screened datasets using the criterion *p* ≤ 0.05 and |logFC| ≥ 2. The results revealed the identification of 182 DEGs in the GSE116250 dataset, comprising 130 up-regulated and 52 down-regulated genes. In the GSE135055 dataset, 171 DEGs were screened, with 121 were up-regulated and 50 were down-regulated. Volcano plots were generated for visualizing the DEGs in both datasets (Fig. 1A and B). Additionally, we generated heatmaps illustrating the top 30 up- and down-regulated genes (Supplementary Tables 3 and 4) from both datasets (Fig. 1C and D). GSEA analysis was performed for all DEGs in the GSE116250 and GSE135055 datasets, respectively, and the three most significant pathways were selected for display (Supplementary Fig. 2).

### Identification of DEGs in HF and functional enrichment analysis

The Venn diagram showed that there were a total of 67 overlapping DEGs in the GSE116250 and GSE135055 datasets, including 51 co-upregulated and 14 co-downregulated genes (Supplementary Fig. 3A-C). To elucidate the biological functions of DEGs, we conducted GO functional annotation and KEGG pathway analyses. The results of the GO analysis demonstrated that the BPs of DEGs were substantially involved in receptor guanylate cyclase signaling pathway, cGMP biosynthetic process, and angiogenesis (Supplementary Fig. 4A). In the CC category, the DEGs were predominantly enriched in extracellular space, extracellular region, and hemoglobin complex (Supplementary Fig. 4B). In the MF category, the DEGs were mainly involved in the hormone receptor binding, hormone activity, and integrin binding (Supplementary Fig. 4C). KEGG pathway enrichment mainly included vascular smooth muscle contraction, cGMP-PKG signaling pathway, and proximal tubule bicarbonate reclamation (Supplementary Fig. 4D).

### Identification of the hub genes

A PPI network of DEGs was established using the STRING database (Supplementary Fig. 5A). Subsequently, hub genes were identified using the cytoHubba plugin in the Cytoscape software (Supplementary Fig. 5B). The top 16 genes were screened out based on their scores (Supplementary Table 5). After reviewing pertinent literature, six genes were chosen for subsequent studies, including FMOD, NPPB, NPPA, COMP, HAPLN1, and NPPC.

### GO, expression, correlation, and PCA analysis of six key genes

**Fig. 2:**
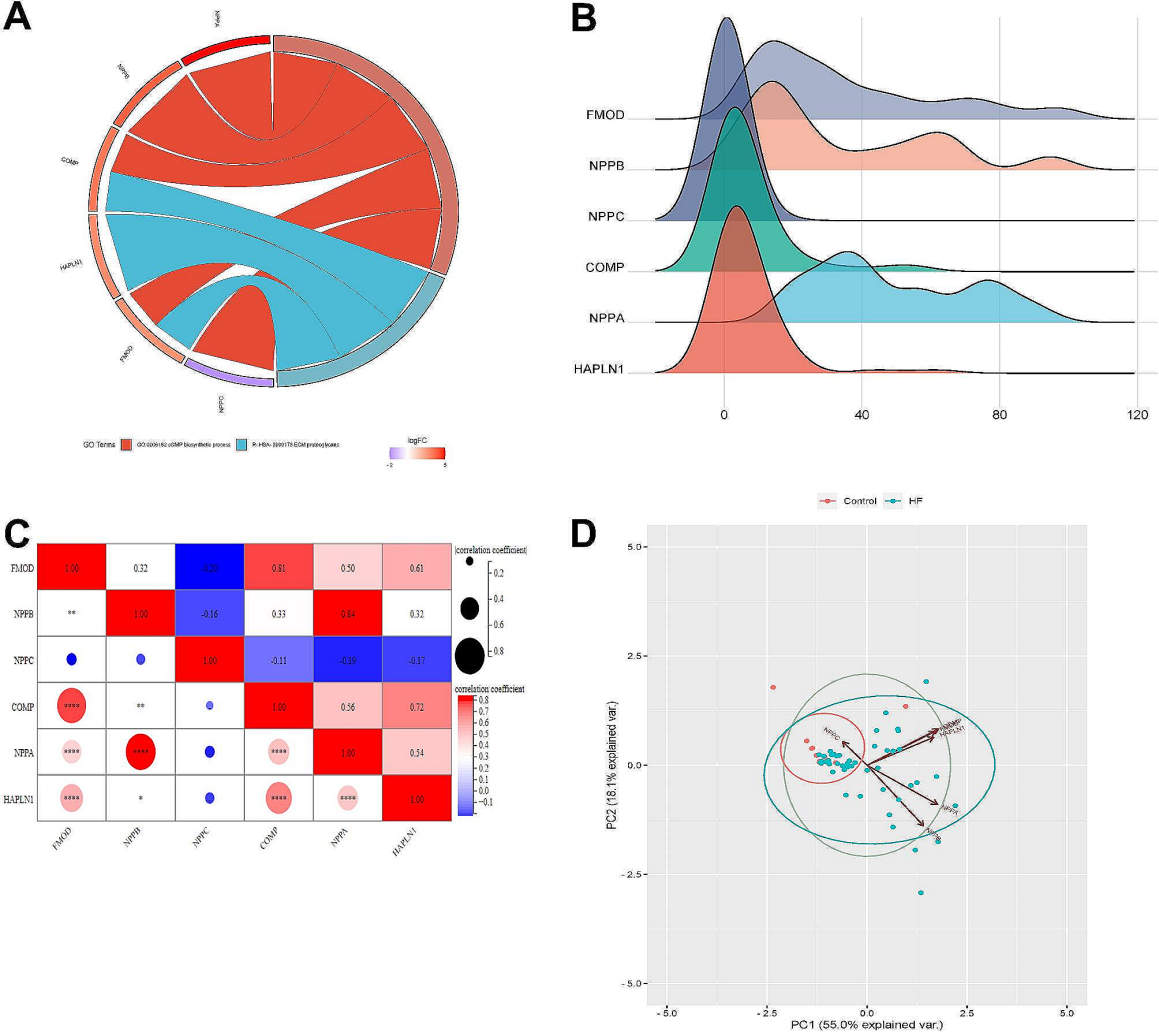
Bioinformatics analysis of key genes. (**A**) Distribution of DEGs for the GO enrichments. (**B**) Ridgeline map of the expression distribution of DEGs. The x-axis corresponds to the gene expression levels, and the y-axis signifies the sample abundance linked to each of these expression levels. (**C**) The correlation heatmap displayed the relationships between six hub DEGs. (**D**) The two principal component variables, PC1 and PC2. PC1 axis explained 55% of the variance, and PC2 accounted for 18.1% variance. Each bioinformatics analysis was performed independently three times. Notes: DEGs, differently expressed genes

Based on the GSE116250 dataset, we performed GO, expression distribution, correlation, and PCA analyses of the six key genes. GO enrichment analysis revealed significant associations of the six key genes with the cGMP biosynthetic process and ECM proteoglycans pathways (Fig. 2A). Gene expression ridgeline maps showed that FMOD, NPPB, and NPPA had relatively high expression levels, with FMOD exhibiting the most abundant expression (Fig. 2B). Correlation analysis revealed significant positive correlations between FMOD and COMP, as well as between NPPB and NPPA (Fig. 2C). Additionally, the PCA analysis yielded two axes, namely PC1 and PC2, where the PC1 axis explained 55% of the variance, while PC2 accounted for only 18.1% of the variance (Fig. 2D).

### HAPLN1 could be used as a diagnostic biomarker for HF

**Fig. 3:**
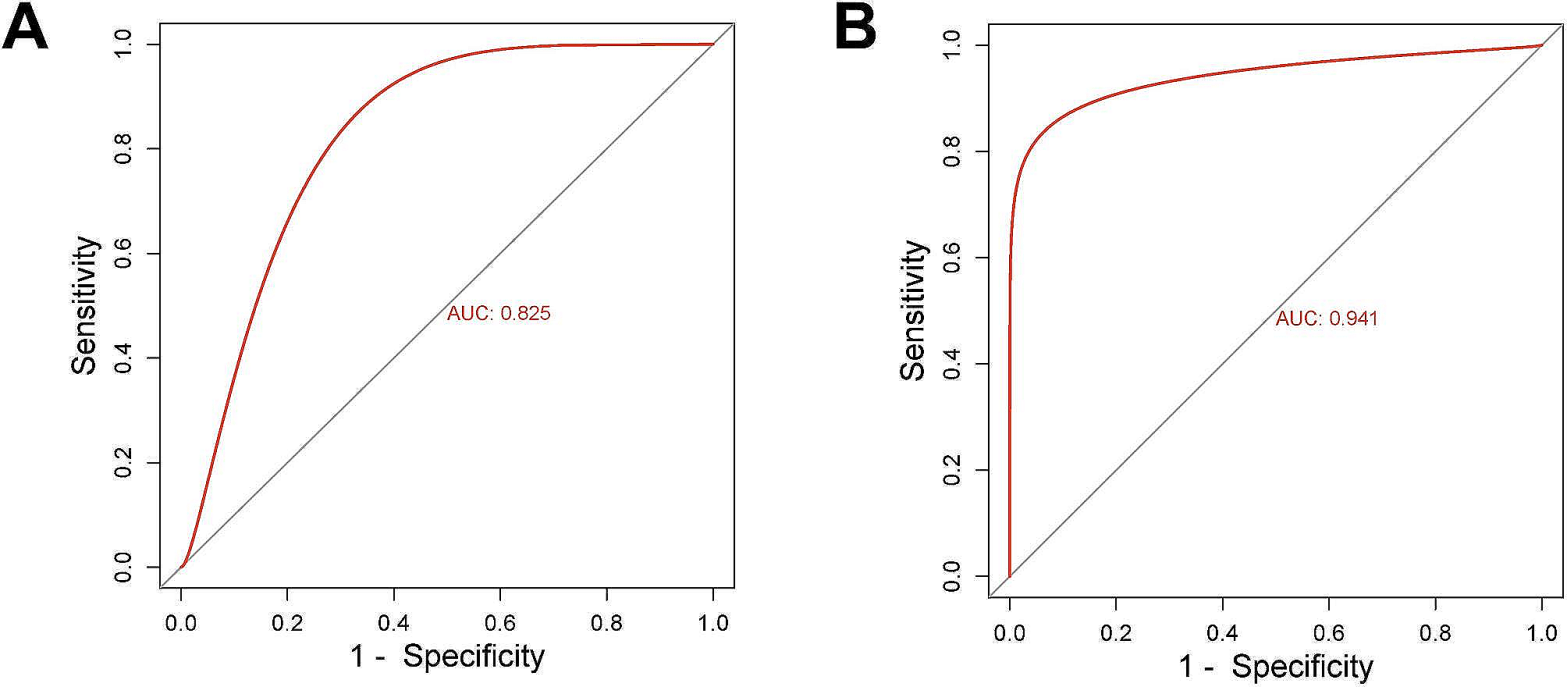
HAPLN1 could be used as a diagnostic biomarker for HF. (**A**-**B**). ROC curve for HAPLN1 in the GSE116250 (AUC = 0.825) and GSE135055 (AUC = 0.941) datasets. Each bioinformatics analysis was performed independently three times. *Notes* HF, heart failure; ROC curve, receiver operating characteristic (ROC) curve; AUC, area under the curve

The diagnostic performance of HAPLN1 was evaluated by the ROC curve, with an area under the curve (AUC) value greater than 0.7 indicating high diagnostic efficiency. The results demonstrated that the AUC of HAPLN1 was 0.825 in the GSE116250 dataset, and in the GSE135055 dataset, it was 0.941(Fig. 3A and B), which suggested that HAPLN1 could differentiate between HF patients and controls.

### Validation of the expression of six hub genes by RT-qPCR in HF cell model

**Fig. 4:**
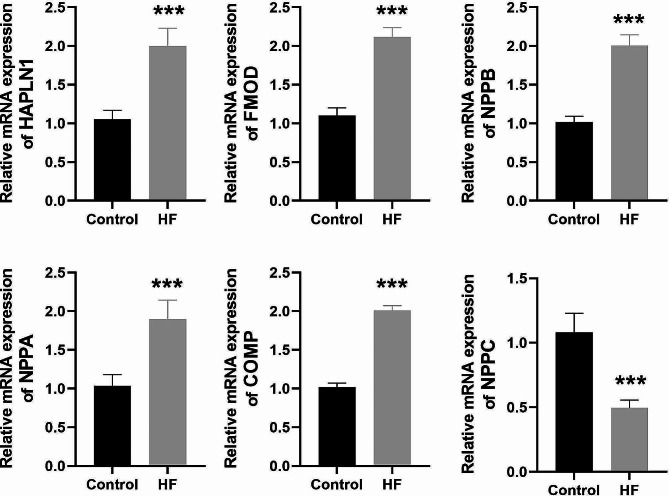
RT-qPCR showed that the expression levels of HAPLN1, FMOD, NPPB, NPPA, and COMP were elevated and NPPC expression level was reduced in the Ang II-induced AC16 cell model (*n* = 3). The results are presented as the mean ± SD from three independent experiments. ^***^P < 0.001 vs. Control group. *Notes* RT-qPCR, Real-time reverse transcriptase-polymerase chain reaction

The human cardiomyocyte cell line (AC16 cells) was induced by Ang II to construct the HF model in vitro. The results demonstrated that, when compared with the Control group, the expression levels of HAPLN1, FMOD, NPPB, NPPA, and COMP were significantly increased (*P* < 0.001), while the expression level of NPPC was significantly decreased in the HF group (*P* < 0.001) (Fig. 4). These findings were consistent with the bioinformatics analysis. Since the function and mechanism of HAPLN1 were fewer reports in HF among the six key genes, along with its potential as a diagnostic biomarker and elevated expression in HF, we chose HAPLN1 for the follow-up study.

### Silencing HAPLN1 promoted HF cell viability, reduced apoptosis, and inhibited cardiac hypertrophy and oxidative stress

**Fig. 5:**
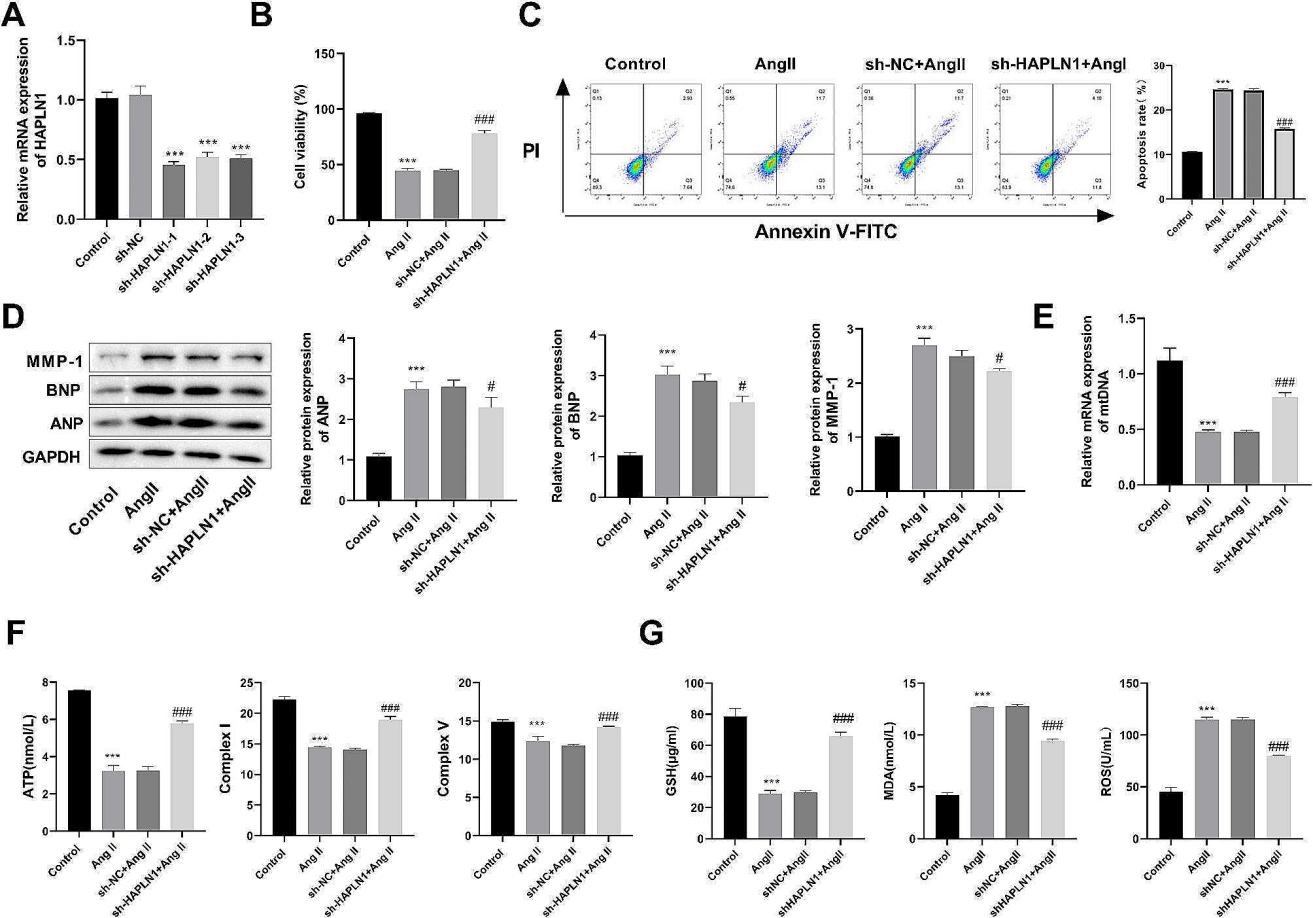
Silencing HAPLN1 promoted HF cell viability, reduced apoptosis, and inhibited cardiac hypertrophy and oxidative stress in Ang II-induced AC16 cells (*n* = 3). (**A**) The efficiency of HAPLN1 silencing was assessed by RT-qPCR, and sh-HAPLN1-1 exhibited the highest knockdown efficiency. (**B**) AC16 cell viability was reduced by Ang II treatment and reversed by HAPLN1 silencing as assessed by CCK8. (**C**) HAPLN1 knockdown reduced apoptosis in Ang II-induced AC16 cells. (**D**) Western blot illustrated that the protein expressions of cardiomyocyte hypertrophy -associated protein (ANP, BNP, and MMP-1) was reduced after HAPLN1 knockdown in Ang II-induced AC16 cells. (**E**) Ang II treatment decreased mtDNA content, whereas knockdown of HAPLN1 promoted mtDNA expression as measured by RT-qPCR. (**F**) ELISA assay showed that mitochondrial ATP generation as well as the activities of complex I and V were decreased in the Ang II group and were increased in the sh- HAPLN1 + Ang II group. (**G**) HAPLN1 knockdown promoted GSH levels and reduced MDA and ROS levels in the Ang II-induced AC16 cells, which were assessed by ELISA assay The results are presented as the mean ± SD from three independent experiments. ^***^P < 0.001 vs. Control group; ^#^*P* < 0.05, ^###^*P* < 0.001 vs. sh-NC + Ang II group. *Notes* HF, heart failure; RT-qPCR, Real-time reverse transcriptase-polymerase chain reaction; CCK8, cell counting kit-8; ELISA, enzyme-linked immunosorbent assay

To provide a more comprehensive understanding of the involvement of HAPLN1 in the development of HF, we employed shRNA to reduce HAPLN1 expression in AC16 cell line. RT-qPCR was used to confirm the knockdown efficiency of HAPLN1, and the results revealed that HAPLN1 was substantially reduced by si-HAPLN1-1, si-HAPLN1-2, and si-HAPLN1-3 (*P* < 0.001) (Fig. 5A). Due to its the most significant knockdown efficiency, si-HAPLN1-1 was selected for subsequent experiments. Then the cells were divided into four groups, including Control, Ang II, sh-NC + Ang II, and sh-HAPLN1 + Ang II groups. The results obtained from CCK-8 indicated that cell viability was notably reduced in the Ang II group when compared to the Control group (*P* < 0.001). In contrast, the viability of AC16 cells in the sh-HAPLN1 + Ang II group was higher than that in the sh-NC + Ang II group (*P* < 0.001) (Fig. 5B). Additionally, Flow cytometry was employed to assess the apoptosis rate. The findings indicated that the apoptosis rate in the Ang II group was elevated compared to the Control group (*P* < 0.001). There is no difference between the sh-NC + Ang II group and the Ang II group (*P* > 0.05). Furthermore, silencing HAPLN1 reduced apoptosis of Ang II-induced AC16 cells (*P* < 0.001) (Fig. 5C).

Then, the levels of cardiomyocyte hypertrophy-related proteins were assessed via the western blot analysis. The outcomes revealed a significant increase in the expression of ANP, BNP, and MMP-1 in the Ang II group compared to the Control group (*P* < 0.001). Notably, there was no significant difference between the sh-NC + Ang II group and the Ang II group (*P* > 0.05). Conversely, the sh-HAPLN1 + Ang II group exhibited reduced expression levels of ANP, BNP, and MMP-1 (*P* < 0.05) in comparison to the sh-NC + Ang II group (Fig. 5D). We detected mtDNA levels, and ATP production and complex I and V activities by RT-qPCR and ELISA, respectively. The results showed that Ang II induction reduced the levels of mtDNA and ATP in AC16 cells (*P* < 0.001). In addition, mitochondrial complex I and complex V activities were also decreased (*P* < 0.001). These trends were reversed by silencing HAPLN1 (*P* < 0.001) (Fig. 5E and F). Furthermore, compared to the Control group, GSH levels were reduced, while MDA and ROS levels were increased in the Ang II group (*P* < 0.001). Knockdown HAPLN1 decreased MDA and ROS levels, while promoting GSH levels (*P* < 0.001) (Fig. 5G). In conclusion, silencing HAPLN1 significantly promoted Ang II-induced AC16 cellular viability, reduced apoptosis, inhibited cardiac hypertrophy and oxidative stress.

### Silencing HAPLN1 activated the PKA signaling pathway in HF cell model

**Fig. 6:**
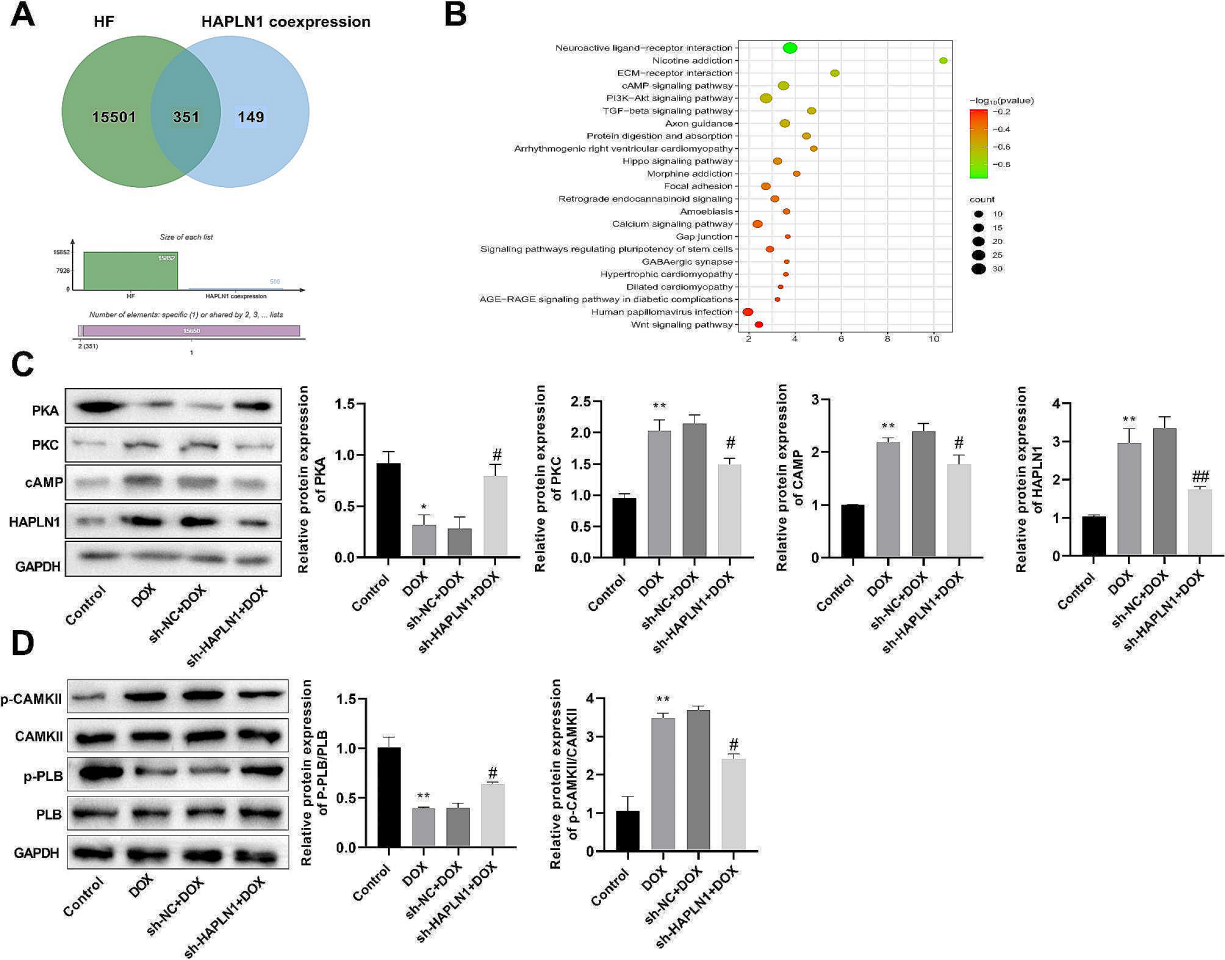
Silencing of HAPLN1 activated the PKA signaling pathway in DOX-induced AC16 cells (*n* = 3). (**A**) The Venn diagram illustrated the 351 genes common to HAPLN1 co-expressed genes and those associated with HF. (**B**) Bubble plot of KEGG enrichment analysis of 351 shared genes. (**C**) Western blot revealed that the protein expressions of PKA pathway-associated protein PKA was increased, while PKC and cAMP were decreased after HAPLN1 knockdown in DOX-induced AC16 cells. (**D**) HAPLN1 silencing reduced protein phosphorylation of CAMKII and promoted protein phosphorylation of PLB in DOX-induced AC16 cells. The results are presented as the mean ± SD from three independent experiments. ^***^P < 0.001 vs. Control group; ^#^*P* < 0.05, ^###^*P* < 0.001 vs. sh-NC + DOX group. *Notes* DOX, doxorubicin

Next, we explored the potential mechanisms underlying the impact of HAPLN1 on the progression of HF. A total of 15,852 genes related to HF were obtained from the Genecards database. Furthermore, 500 genes co-expressed with HAPLN1 were collected from the Coxpresdb database (Fig. 6A). Venn diagram revealed that there were 351 overlapping genes, mainly enriched in the cAMP, PI3K-Akt, and TGF-β signaling pathways (Fig. 6B). Previous studies have shown that the cAMP/PKA signaling pathway plays an important role in HF [25]. Then the effect of knocking down HAPLN1 on the expression levels of key proteins in the cAMP/PKA signaling pathway was examined by western blot. The results showed that protein expression levels of HAPLN1(*P* < 0.01), cAMP (*P* < 0.01), and PKC (*P* < 0.01) were up-regulated in the DOX group compared with the Control group, while PKA (*P* < 0.05) was significantly down-regulated, which was reversed by silencing HAPLN1 (*P* < 0.05) (Fig. 6C). Moreover, DOX treatment decreased the ratio of p-PLB/PLB and increased the ratio of p-CAMKII/CAMKII (*P* < 0.01). Conversely, the knockdown of HAPLN1 reversed this trend (*P* < 0.05) (Fig. 6D). These above results suggested that silencing HAPLN1 activated the PKA signaling pathway.

### Construction of HF rat model

**Fig. 7:**
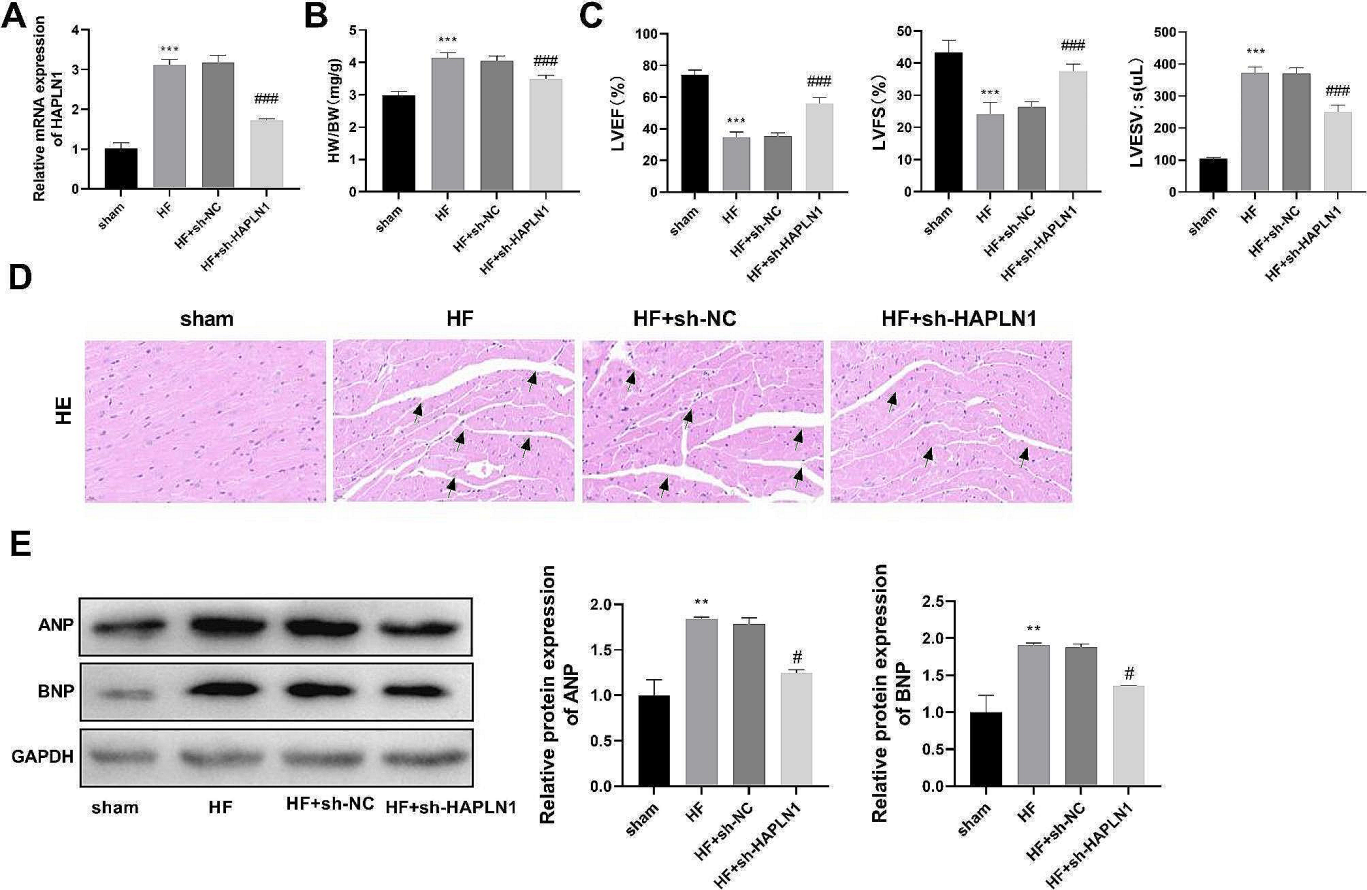
Construction of the HF animal model using ISO (*n* = 6). (**A**). HAPLN1 was efficiently knocked down in the ISO-induced rat models as detected by RT-qPCR. (**B**-**C**). HAPLN1 knockdown improved cardiac function indicators in the HF rat model, including HW/BW, LVFS, LVEF, and LVESV. (**D**). HE staining results showed that HAPLN1 knockdown ameliorated the pathologic changes in cardiac tissue in the HF rat model. Scale bar = 50 μm. Black arrows represent irregular cross-striations of myocardial tissue. (**E**). Protein expression levels of ANF and BNF were elevated in the HF rat model, which was significantly reversed by HAPLN1 knockdown. The results are presented as the mean ± SD from three independent experiments. ^**^P < 0.01, ^***^P < 0.001 vs. sham group; ^#^*P* < 0.05, ^###^*P* < 0.001 vs. HF + sh-NC group. *Notes*: ISO, isoproterenol; RT-qPCR, Real-time reverse transcriptase-polymerase chain reaction; HW/BW, heart weight/body weight; LVEF, left ventricular ejection fraction; LVFS, left ventricular fraction shortening; LVESV, left ventricular end-systolic volume; HE staining, hematoxylin and eosin (HE) staining

To further explore the function and mechanism of HAPLN1, we constructed the rat model of HF with HAPLN1 knockdown. RT-qPCR results showed that HAPLN1 expression was significantly elevated in the HF group compared with that in the sham group (*P* < 0.001). In contrast, HAPLN1 expression in the HF + sh-HAPLN1 group was notably lower than that in the HF + sh-NC group (*P* < 0.001) (Fig. 7A). Compared with the sham group, the HW/BW index of rats in the HF group was obviously elevated, suggesting that the rats developed cardiac hypertrophy. After knockdown of HAPLN1, the HW/BW index was significantly reduced (*P* < 0.001) (Fig. 7B). Additionally, cardiac function index tests revealed significantly lower LVFS and LVEF as well as significantly higher LVESV in the HF group compared to that in the sham group (*P* < 0.001). Compared with the HF-sh-NC group, LVFS and LVEF were notably elevated in the HF + sh-HAPLN1 group, whereas LVESV was significantly reduced (*P* < 0.001) (Fig. 7C). HE staining revealed that cardiomyocytes in the sham group exhibited orderly arrangement, normal morphology, with clear nuclei and horizontal stripes, and no obvious pathological damage. In contrast, cardiomyocytes in the HF group and HF + sh-NC group displayed disorganization, hypertrophy, with more broken and dissolved fibers, and evident inflammatory infiltration. However, in the HF + sh-HAPLN1 group, cardiac tissue damage was notably improved, characterized by clearer cross-section, more orderly arrangement of cells, and only a minor amount of inflammatory infiltration comparison with the HF + sh-NC group (Fig. 7D). Western blot analysis for cardiac hypertrophic proteins ANP and BNP revealed a significant increase in their levels in the HF group compared to that in the sham group (*P* < 0.01). Knockdown of HAPLN1 greatly inhibited the protein levels of ANP and BNP (*P* < 0.05) (Fig. 7E).

### Silencing HAPLN1 activated the PKA signaling pathway in HF rat model

**Fig. 8:**
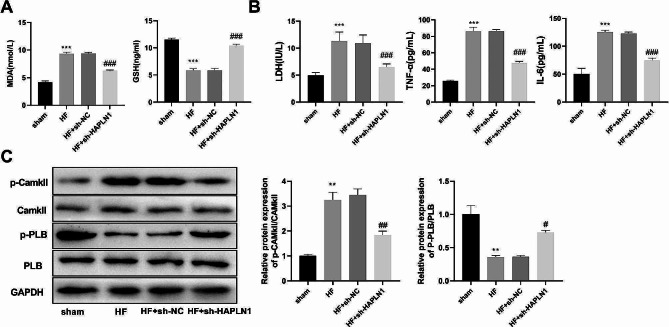
Silencing HAPLN1 activated the PKA signaling pathway in HF rat model (*n* = 6). (**A**) HAPLN1 knockdown reduced MDA level and elevated GSH level in the HF rat model. (**B**) HAPLN1 knockdown inhibited the expression of apoptosis-related factors, including LDH, TNF-α, and IL-6 in the HF rat model. (**C**) In the HF rat model, silencing HAPLN1 reduced CAMKII protein phosphorylation and promoted PLB protein phosphorylation. The results are presented as the mean ± SD from three independent experiments. ^**^P < 0.01, ^***^P < 0.001 vs. sham group; ^#^*P* < 0.05, ^##^*P* < 0.01, ^###^*P* < 0.001 vs. HF + sh-NC group

Then, the levels of MDA and GSH in the serum of rats in each group were detected by ELISA. Higher level of MDA and lower level of GSH were observed in the HF group compared to that in the sham group (*P* < 0.001). Compared with the HF + sh-NC group, MDA expression was notably reduced, while the level of GSH was significantly elevated in the HF + sh-HAPLN1 group (*P* < 0.001) (Fig. 8A). Furthermore, apoptosis-related factors were examined in rat serum. The levels of LDH, TNF-α, and IL-6 were significantly increased in the HF group compared with that in the sham group (*P* < 0.001). Silencing of HAPLN1 notably inhibited the secretion of these factors (*P* < 0.001) (Fig. 8B). Furthermore, western blot showed that the expression of p-CAMKII/CAMKII was substantially elevated, whereas the expression of p-PLB/PLB was significantly reduced in the HF group compared to that in the sham group, which was considerably reversed by the HAPLN1 knockdown (*P* < 0.05) (Fig. 8C).

## Discussion

HF remains a significant contributor to mortality, morbidity, and compromised quality of life [26]. With the substantial aging of the population, HF has emerged as a noteworthy social and public health concern [27]. Therefore, it is crucial to explore the underlying molecular mechanisms of HF. In this study, we identified six key genes associated with HF by analyzing the GSE116250 and GSE135055 datasets. The silencing of HAPLN1 resulted in an enhancement of HF cell viability, a suppression of apoptosis, a reduction in the cardiomyocyte hypertrophy and oxidative stress through the PKA signaling pathway activation.

With the rapid advancement of high-throughput technology and bioinformatics, an increasing number of genes are showing promise in the development of HF. Yuan et al. identify five key genes associated with HF through bioinformatics analysis, including FRZB, SFRP4, ENTPPL, AQP4, and C1OF105 [28]. Another study, analyzing the GSE76701, GSE21610, and GSE8331 microarray datasets, discovered that that EIF1AY, RPS4Y1, USP9Y, KDM5D, DDX3Y, NPPA, HBB, TSIX, LOC28556, and XIST are potential new targets for HF [29]. In this study, six key genes were identified based on the GSE116250 and GSE135055 datasets through bioinformatics methods, including HAPLN1, FMOD, NPPB, NPPA, COMP, and NPPC.

Ang II, the main effector of the renin-angiotensin system, binds to the angiotensin type 1 receptor, leading to cardiac fibroblast proliferation, intercellular collagen overexpression, matrix deposition, and myocardial fibrosis [30]. Ang II treatment induces myocardial hypertrophy and enhances the oxidative stress in cardiomyocytes [31]. In this study, AC16 cells induced by Ang II was used to construct HF model in vitro. The findings indicated that Ang II stimulation promoted AC16 cell apoptosis, cardiac hypertrophy, and oxidative stress, which was consistent with previous studies [32, 33]. Subsequently, we explored the expression levels of six key genes in HF cell model. We found that the expression of HAPLN1, FMOD, NPPB, NPPA, and COMP was elevated, while NPPC was decreased in HF. FMOD has consistently emerged as a key gene in HF in several previous bioinformatics studies [34, 35]. Moreover, FMOD is found to be expressed in cardiomyocytes and cardiac fibroblasts in the hearts of HF patients and mice, and its expression was notably upregulated by 3-10-fold due to pro-inflammatory stimulation [36]. NPPA, NPPB, and NPPC are all natriuretic peptides, encoding ANP, BNP, and CNP, respectively [37]. ANP and BNP are primarily synthesized by cardiomyocytes, whereas CNP is more prevalent in the central nervous system and peripheral tissues [38]. Both ANP and BNP possess properties that contribute to vasodilation, increased natriuresis, diuresis, as well as antifibrotic and antihypertrophic effects in the heart [39]. Additionally, ANP and BNP play important roles in regulating energy balance. They enhance the oxidative prowess of skeletal muscles and stimulate lipolysis in subcutaneous adipose tissue [40]. ANP and BNP serve as diagnostic, predictive, and prognostic markers for HF, and they also act as targets for therapeutic interventions [41]. Although CNP primary function is not as a cardiac hormone, it also exerts cardiovascular effects, encompassing activities like re-endothelialization, hyperpolarization, antithrombotic properties, and inhibition of fibrosis [42, 43]. COMP promotes the stability of the ECM network by directly binding to other ECM components, including collagen and TGF-β1, to form collagen fibers [44], which is essential for maintaining cardiac homeostasis. COMP deficiency leads to dilated cardiomyopathy by reducing integrin β1 expression and signaling [45]. Additionally, a study suggests that COMP is a potential biomarker for cardiac fibrosis [46]. Both COMP and HAPLN1 are key components of the ECM, implying that the ECM plays a significant role in the progression of HF.

The cardiac ECM network consists of two main components: an interstitial component that envelops all myocardial cells, providing a structural framework, and a pericellular component that closely interacts with specific cell types [47]. Once initiated, ECM remodeling persists and contributes to both systolic and diastolic functional impairments [48]. There is a compelling correlation between the enlargement of the cardiac ECM and unfavorable outcomes among individuals with HF. In patients experiencing HF with reduced ejection fraction, the extent of fibrosis serves as a predictive indicator for mortality and adverse cardiac events [49]. After initial injury, cardiac fibroblasts are activated and subsequently differentiate into myofibroblasts with proliferative and secretory characteristics that contribute to ECM turnover, collagen deposition, and cardiomyocyte proliferation and differentiation [50]. In a variety of heart diseases, cardiac fibroblasts become dysregulated during myocardial remodeling, leading to a general accumulation of ECM. This process results in cardiac fibrosis and increases the risk of HF in many patients [51]. In the present study, we found that knockdown of HAPLN1, a key gene in the ECM, significantly promoted HF cell viability and reduced apoptosis, cardiac hypertrophy and oxidative stress response, which has not been reported before.

Prior to the development of HF, cardiac hypertrophy and remodeling occurs as a response to internal or external stress. Persistent cardiomyocyte enlargement, on the other hand, leads to pathological cardiac hypertrophy, cardiac fibrosis, and, eventually, HF [52]. In the present investigation, we found that the expression of cardiomyocyte hypertrophy-related proteins, including ANP, BNP, and MMP-1, was significantly elevated in the Ang II-induced HF cell model, which was reversed by knockdown of HAPLN1. Furthermore, it has been demonstrated that continuous exposure of the hypertrophied myocardial to vasoactive hormones causes abnormalities in mitochondrial electron transport, metabolic derangements, and functional hypoxia, which in turn leads to oxidative stress development [53]. Oxidative stress is augmented in hypertrophied myocardium in HF, as has been demonstrated in both experimental and clinical observations [54]. In this study, knockdown of HAPLN1increased mtDNA levels, ATP levels, and the activity of complexes I and V, while reducing MDA and ROS levels. Currently, cardiac hypertrophy and oxidative stress are promising targets for HF therapy [55]. Altogether, in the present study, we found that HAPLN1 knockdown suppressed cardiac hypertrophy and oxidative stress in HF.

Next, we explored the molecular mechanisms by which HAPLN1 affected HF progression. DOX is a highly effective chemotherapeutic drug used to treat solid tumors. Its clinical use and therapeutic value, however, are clearly hampered by the life-threatening cardiotoxicity that eventually causes left ventricular dysfunction and congestive HF [56]. Previous study has shown that meteorin-like protein reduces OXA-induced cardiotoxicity by triggering the cAMP/PKA/SIRT1 signaling pathway [57]. Low-dose Metformin exerts protective effects on cardiomyocytes against DOX-induced cytotoxicity through a sequential engagement of AMPK, PKA/CREB1, Src, and PDGFR [58]. These findings suggested that activation of the PKA/cAMP signaling pathway attenuated DOX-induced cardiotoxicity. Therefore, we subsequently induced AC16 cells by DOX to explore the correlation between HAPLN1 and PKA/cAMP signaling pathways. We found that knocking down HAPLN1 promoted PKA expression in DOX-induced AC16 cells. Moreover, it was also validated in the ISO-induced HF rat model. In addition, knockdown of HAPLN1 increasedp-PLB/PLB ratio and reduced p-CAMKII/CAMKII ratio in vivo and in vitro. Regulation of cAMP-dependent protein kinase (PKA) governs autonomic control of ventricular function. Dysregulated protein kinase signaling is a characteristic feature of ischemic heart disease [59]. During the fight-or-flight response, the swift release of catecholamines significantly elevates heart rate and contractility by triggering the PKA signaling pathway through β-AR-dependent activation [60]. Pacemaker cells exhibit distinct characteristics with elevated basal PKA activity. These attributes are essential and capable of generating rhythmic internal Ca2 + store oscillations and spontaneous beating, even in the absence of β-adrenergic stimulation [61]. Moreover, PKA-dependent phosphorylation of RyR2 and PLB improves the role of RyR2 and SERCA2a, which plays an important role in the systolic function [62]. Reduced sarcoplasmic reticulum Ca2 + concentration and decreased sarco/endoplasmic reticulum Ca2+-ATPase (SERCA) function could result from decreased PLB phosphorylation at the CaMKII site in HF [63]. In summary, it can be concluded that HAPLN1 knockdown potentially impacts HF development by activating the PKA pathway.

However, there are some limitations in this study. First, while we found that knockdown of HAPLN1 inhibited the development of HF, the functions of other key genes have not been elucidated. Second, cell model of HF was limited to AC16 cells, and further investigation is required to confirm the role and potential mechanism of HAPLN1 in other HF cell models. Moreover, our in vivo experiments relied on ISO-induced HF models, which might not entirely capture all facets of human HF pathology. Therefore, additional investigations employing alternative animal models and patient cohorts are needed to validate our findings more comprehensively.

## Conclusions

This study identified two key genes associated with the ECM, including COMP and HAPLN1, implying the significant involvement of ECM in the advancement of HF. HAPLN1 could be used as a diagnostic biomarker for HF. Knockdown of HAPLN1 inhibited the progression of HF, which may be by activating the PKA signaling pathway.

### Electronic supplementary material

Below is the link to the electronic supplementary material.

Supplementary Material 1

Supplementary Material 2

Supplementary Material 3

Supplementary Material 4

## Data Availability

The datasets used and analyzed during the current study are available from the corresponding author on reasonable request. Accession numbers of the datasets used in current study are GSE116250 and GSE135055 in Gene Expression Omnibus.
