# Supplementary material for: HAPLN1 knockdown inhibits heart failure development via activating the PKA signaling pathway
Source: BMC Cardiovasc Disord. 2024 Apr 5;24:197. doi: 10.1186/s12872-024-03861-8 (PMC10996236; doi:10.1186/s12872-024-03861-8)

Fig7D GAPDH

include multiple exposures

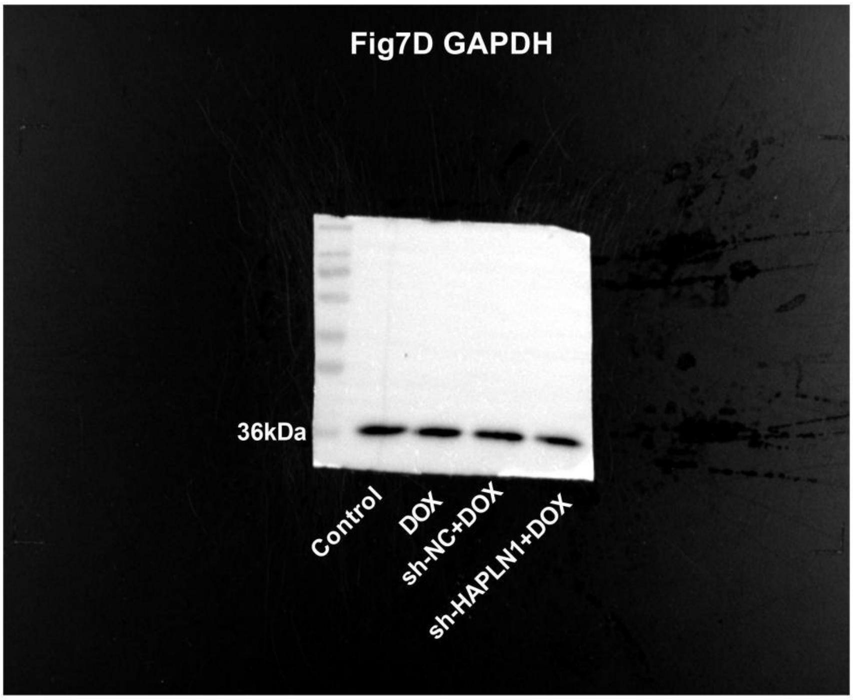

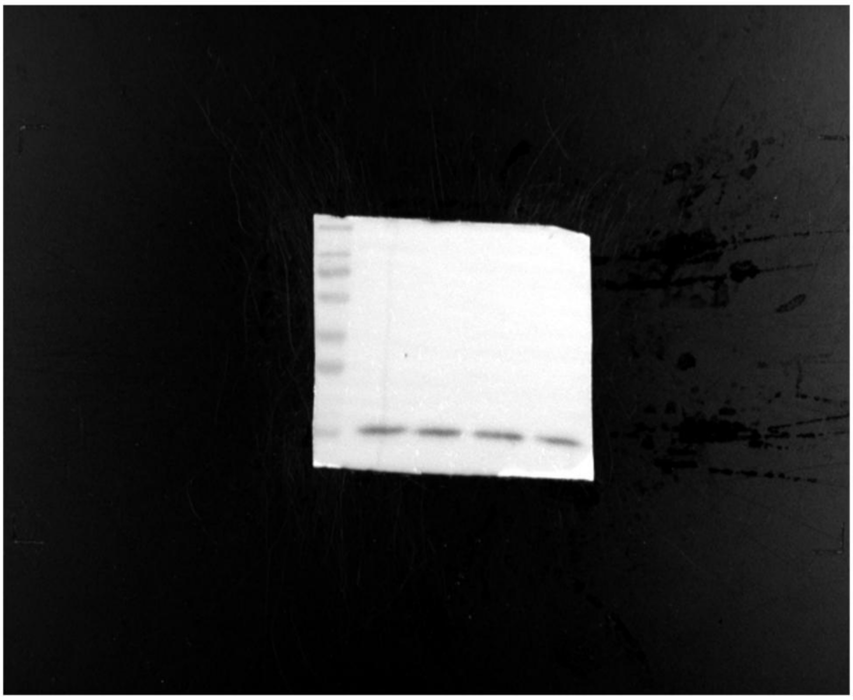

Fig7D ANP

include multiple exposures

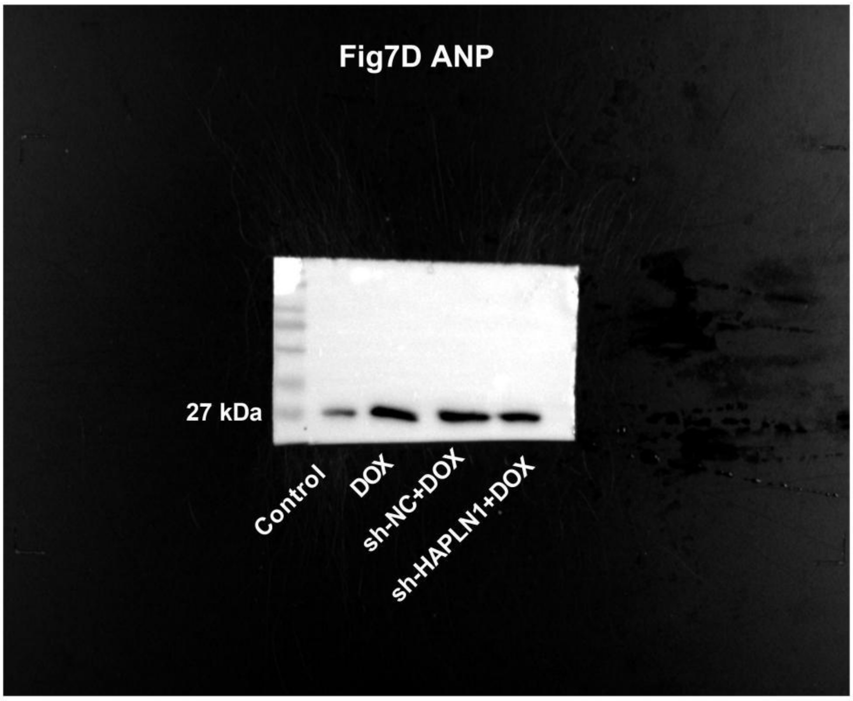

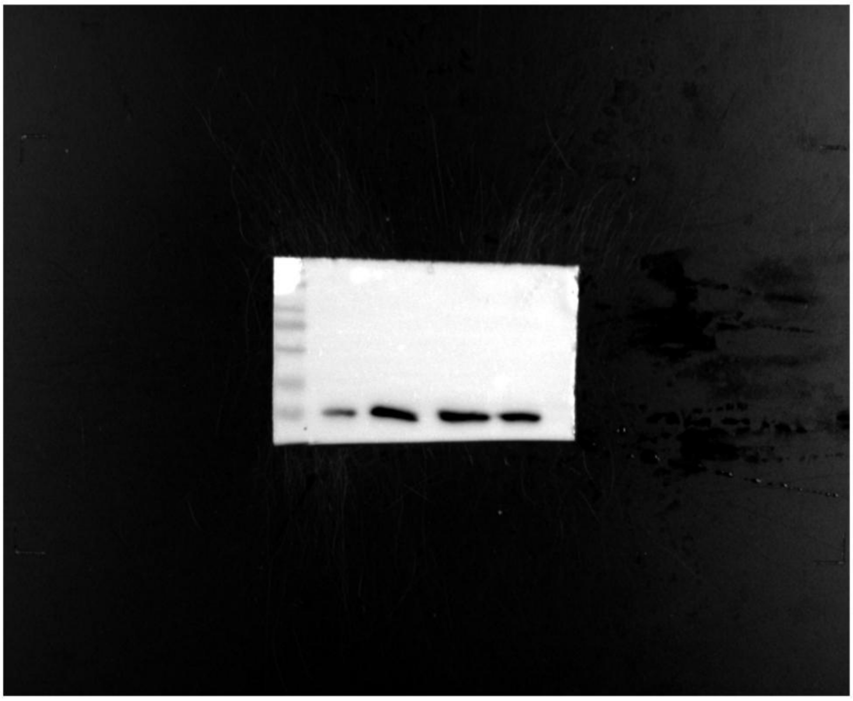

Fig7D BNP

include multiple exposures

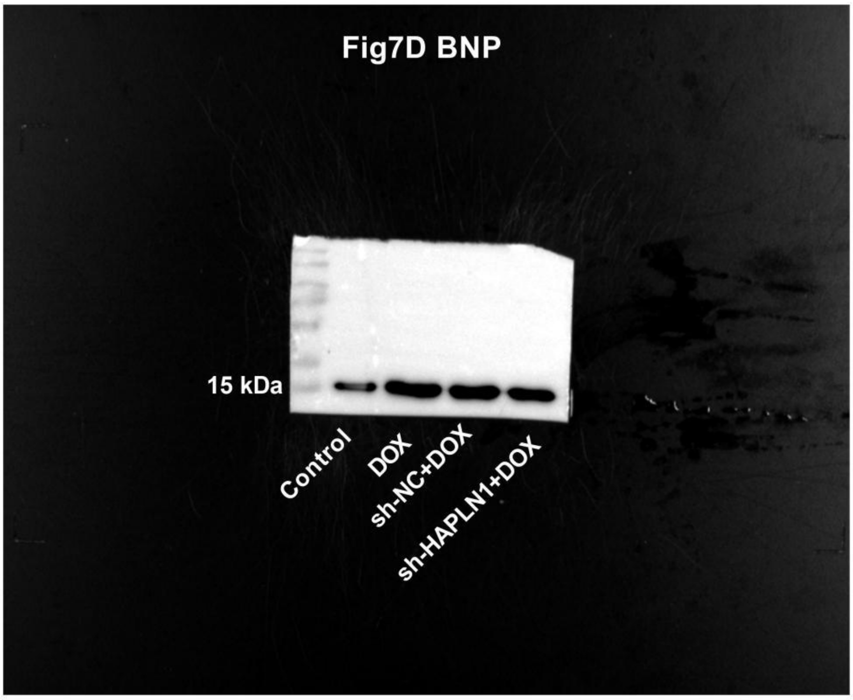

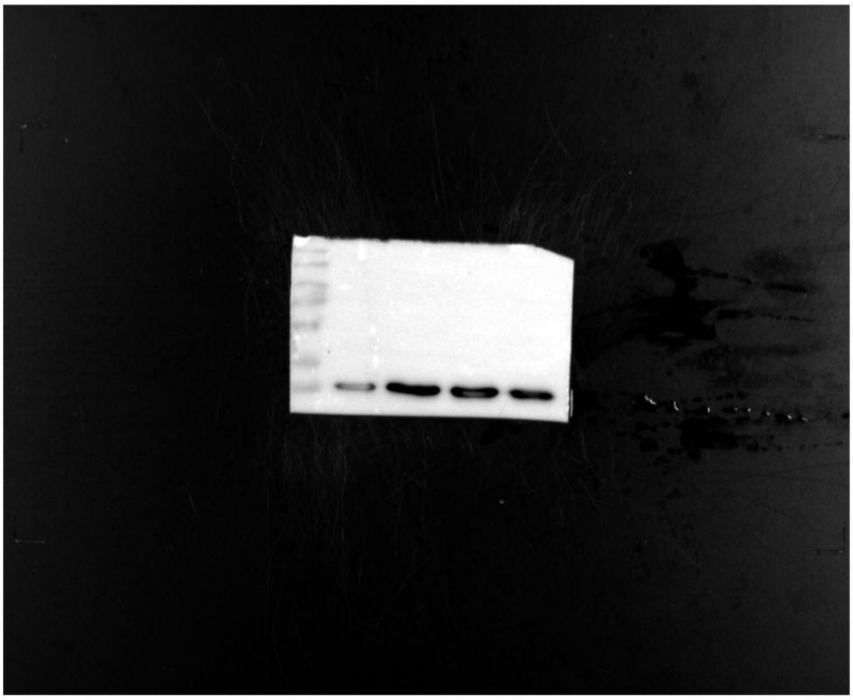

Fig7D MMP-1

include multiple exposures

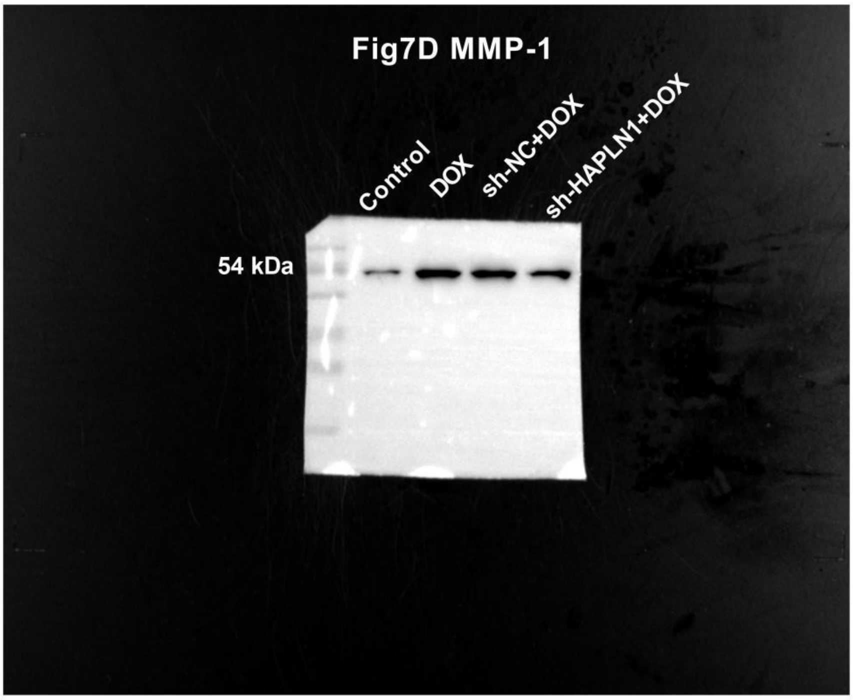

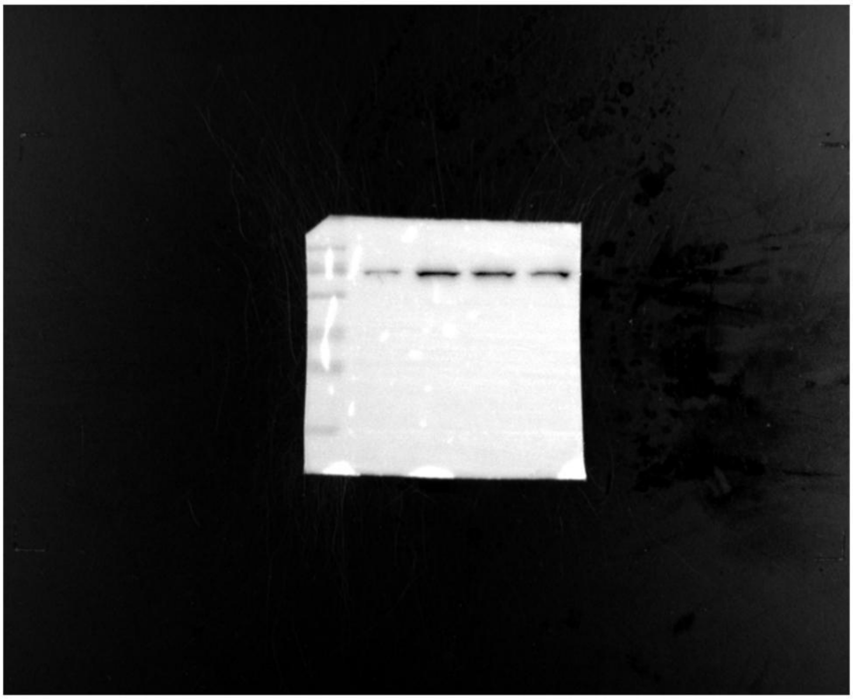

Supplement: Supplementary file 1 — Supplementary Material 1 [file 12872_2024_3861_MOESM1_ESM.pdf]
