# Supplementary material for: HAPLN1 knockdown inhibits heart failure development via activating the PKA signaling pathway
Source: BMC Cardiovasc Disord. 2024 Apr 5;24:197. doi: 10.1186/s12872-024-03861-8 (PMC10996236; doi:10.1186/s12872-024-03861-8)

Fig8C GAPDH

include multiple exposures

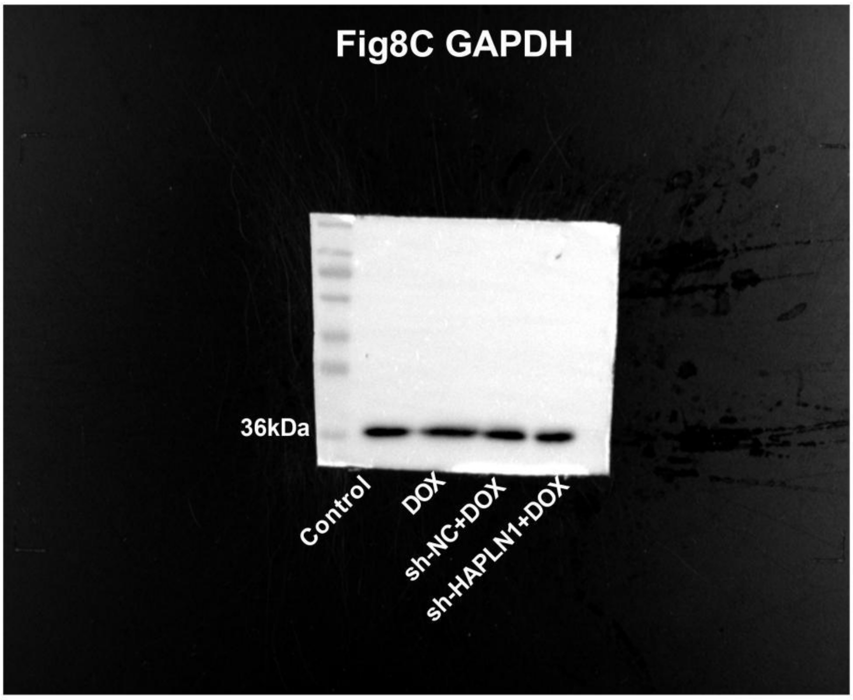

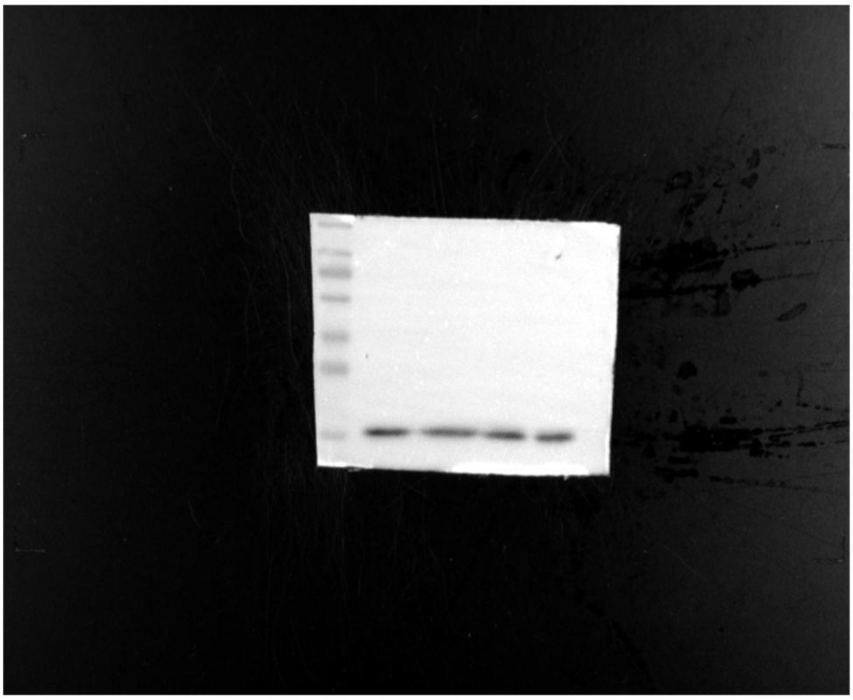

Fig8C PKC

include multiple exposures

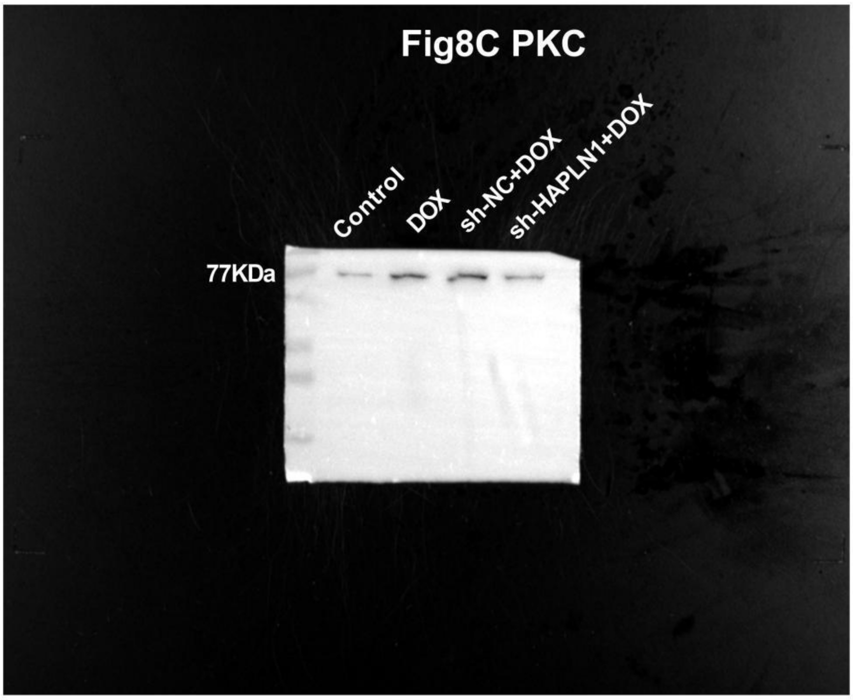

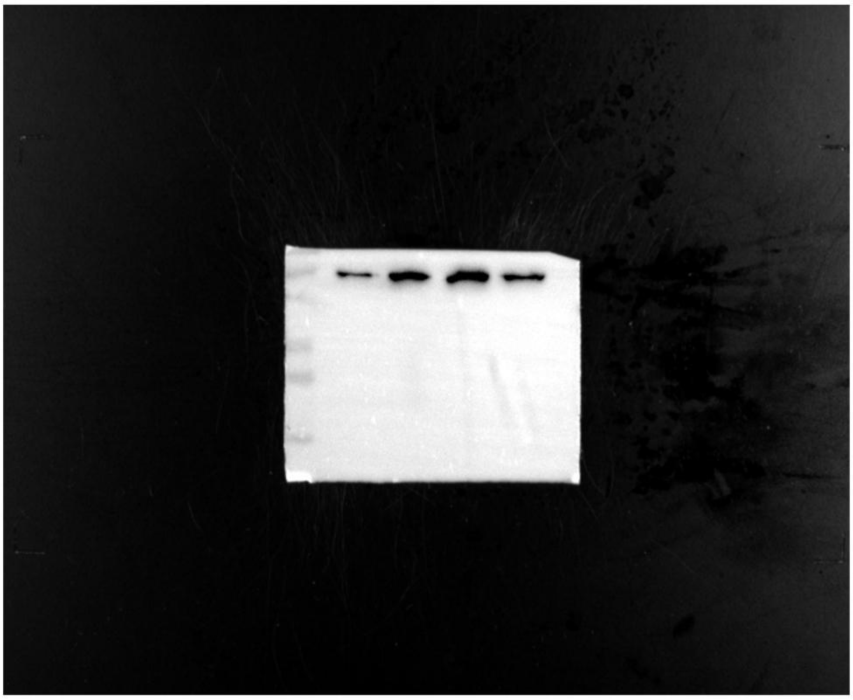

Fig8C PKA

include multiple exposures

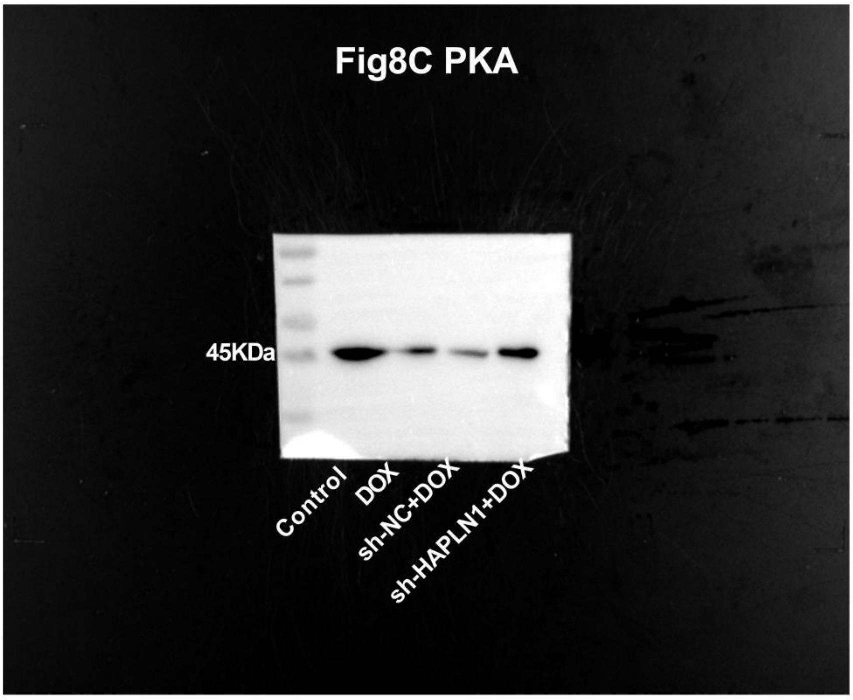

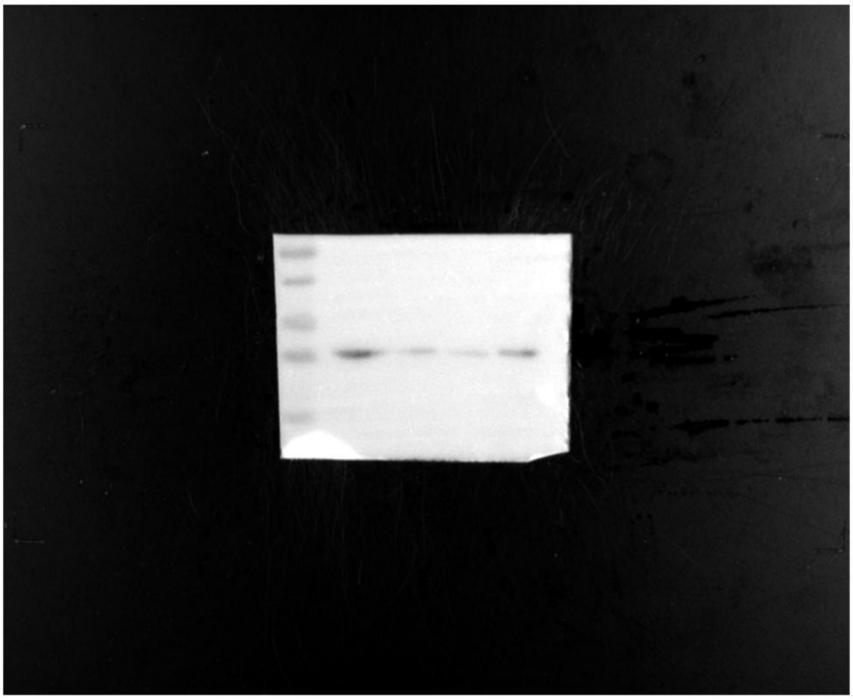

Fig8C cAMP

include multiple exposures

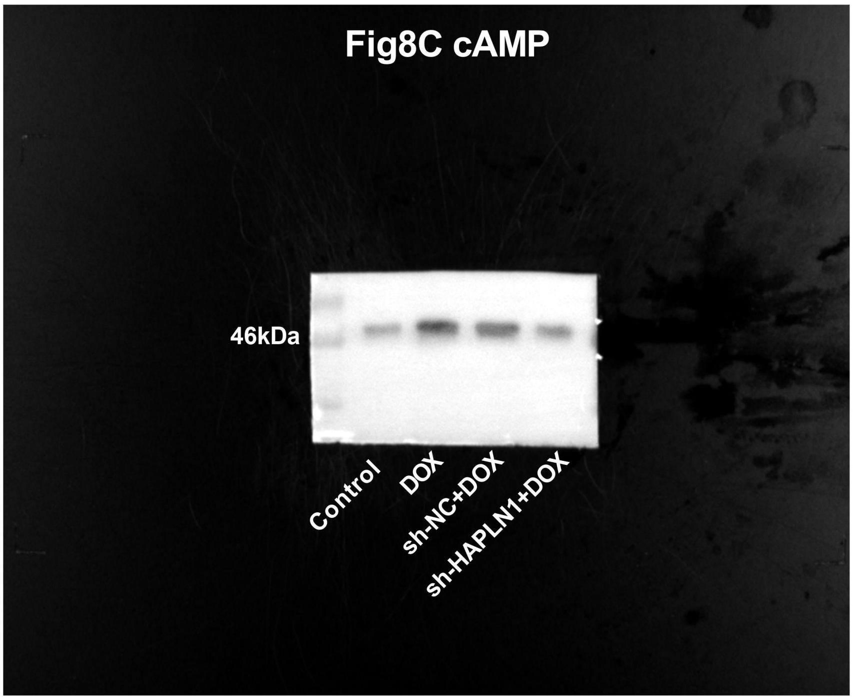

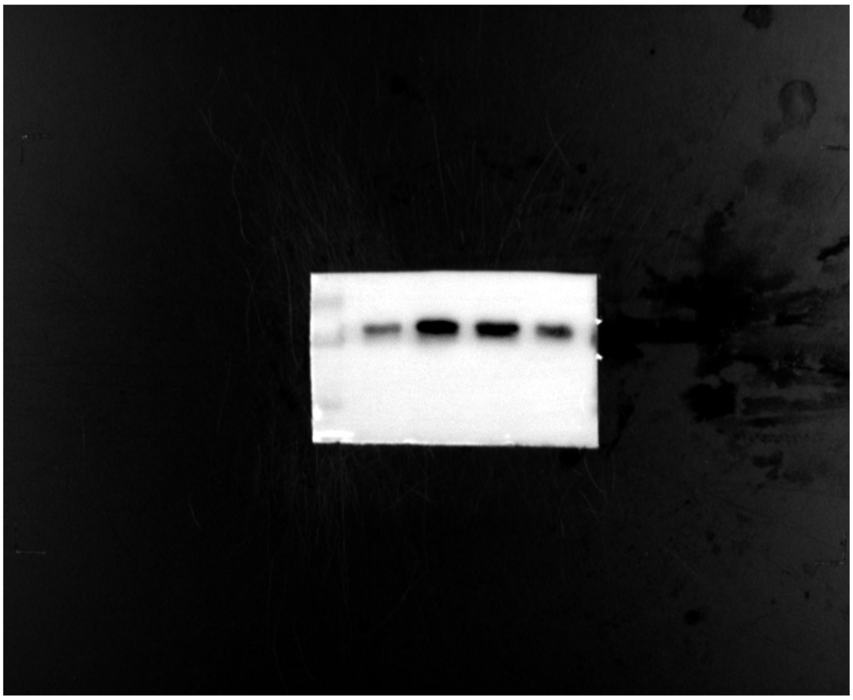

Fig8C HAPLN1

include multiple exposures

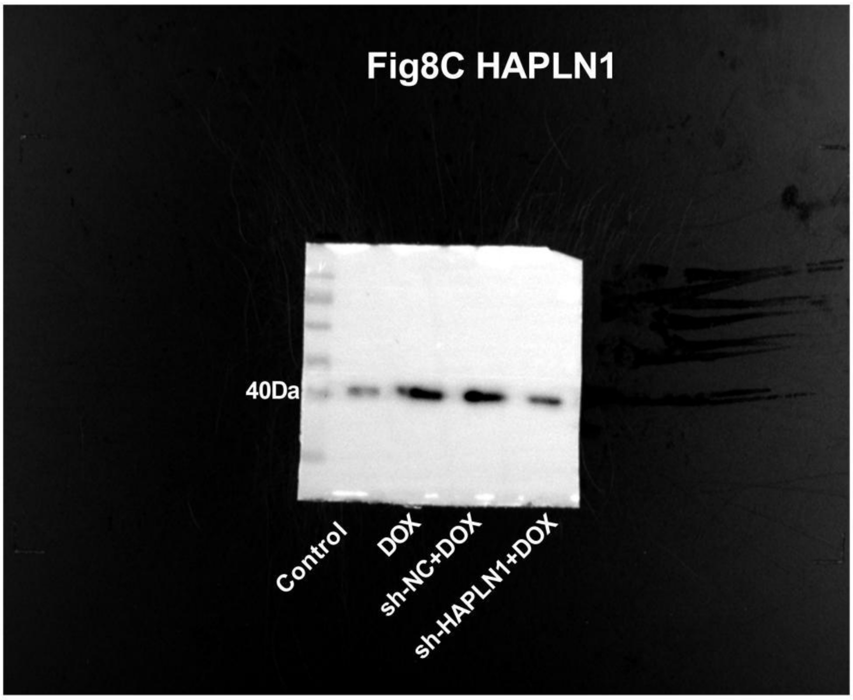

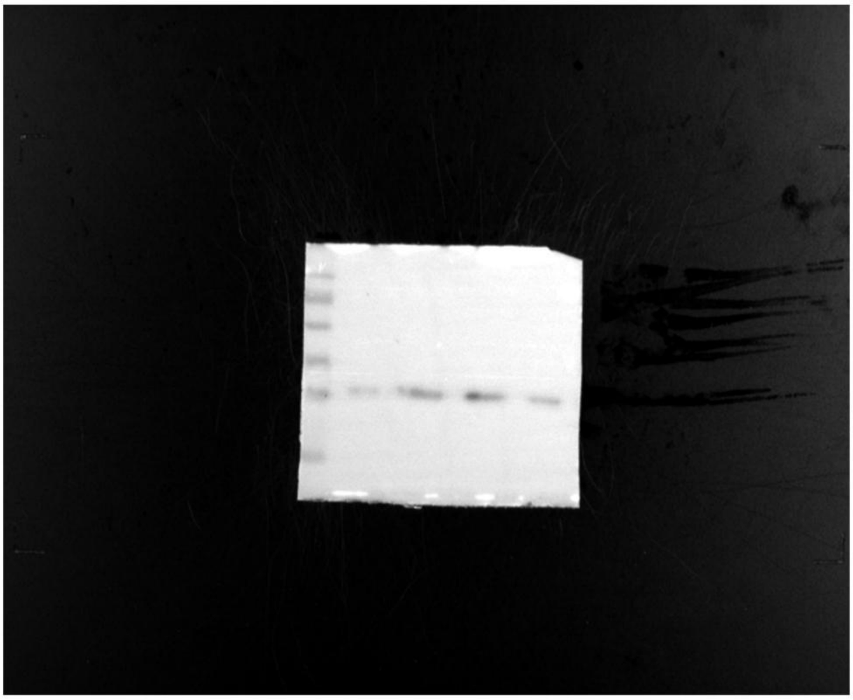

Supplement: Supplementary file 2 — Supplementary Material 2 [file 12872_2024_3861_MOESM2_ESM.pdf]
