# Supplementary material for: HAPLN1 knockdown inhibits heart failure development via activating the PKA signaling pathway
Source: BMC Cardiovasc Disord. 2024 Apr 5;24:197. doi: 10.1186/s12872-024-03861-8 (PMC10996236; doi:10.1186/s12872-024-03861-8)

Fig8D GAPDH-include multiple exposures

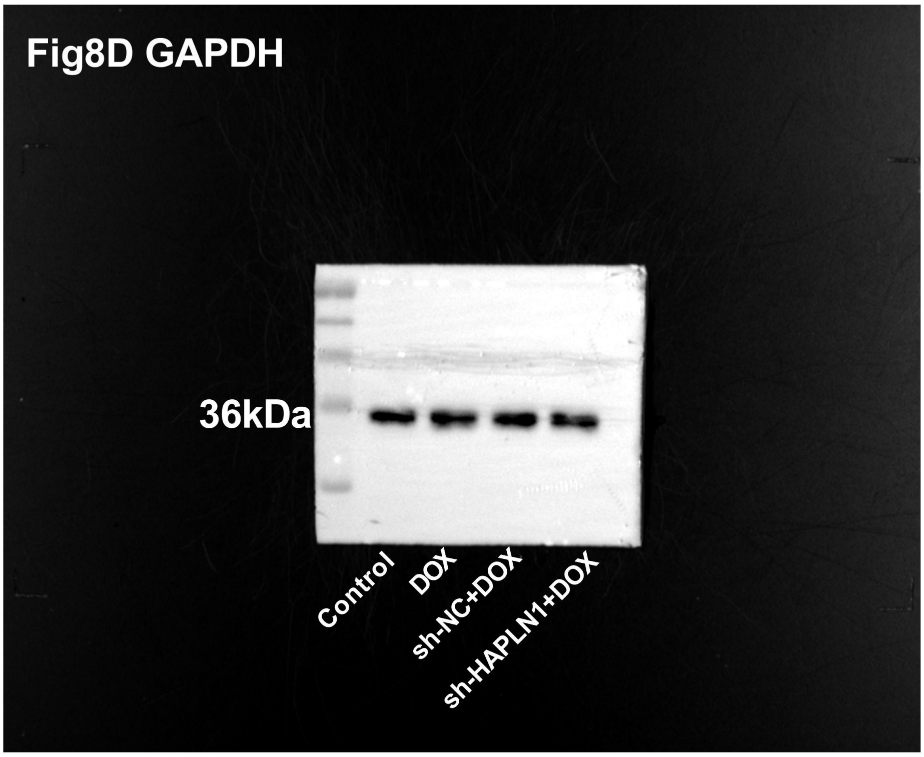

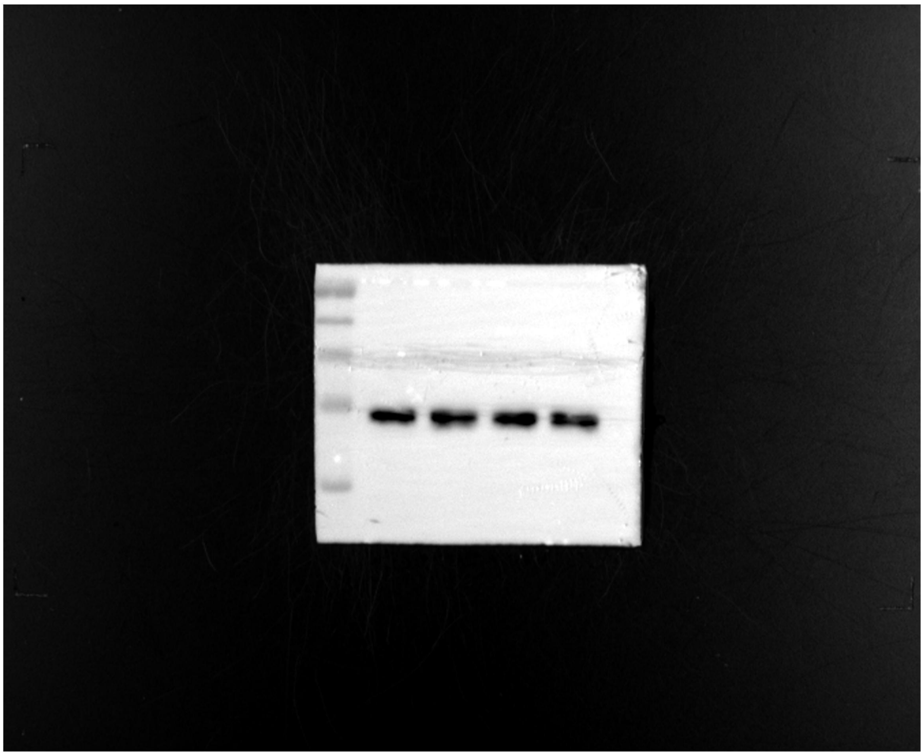

Fig8D PLB-include multiple exposures

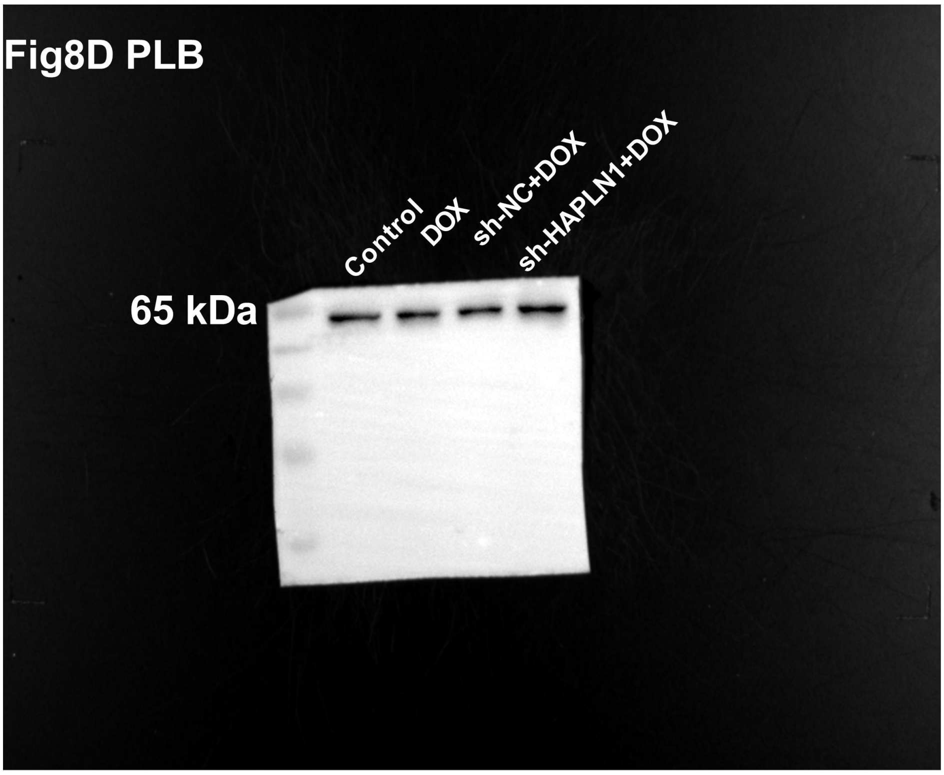

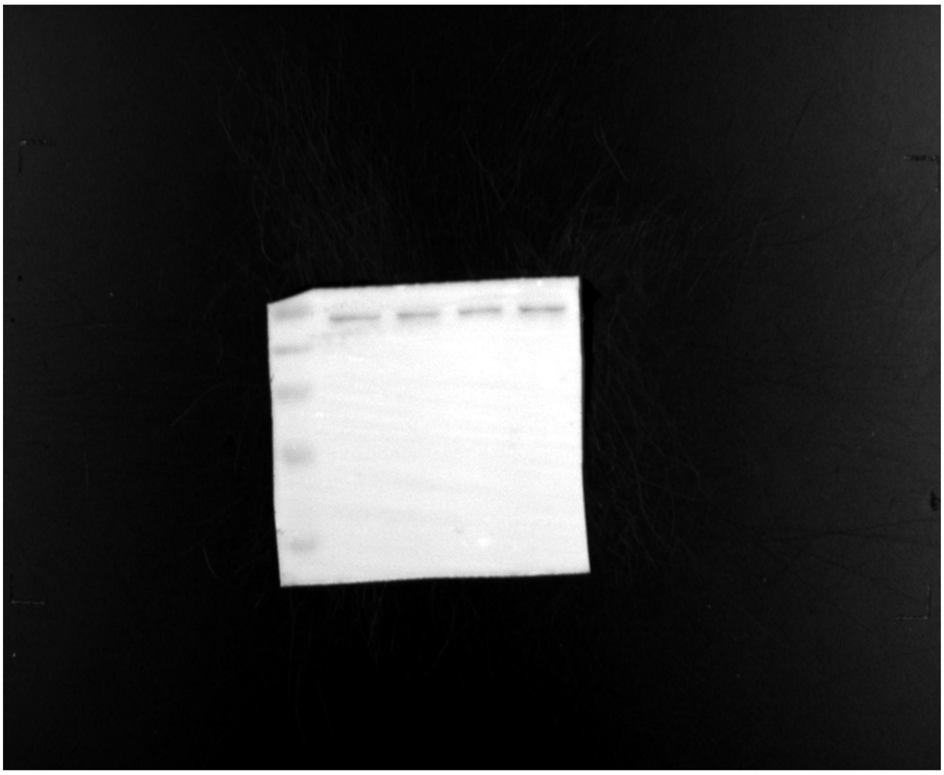

Fig8D P-PLB-include multiple exposures

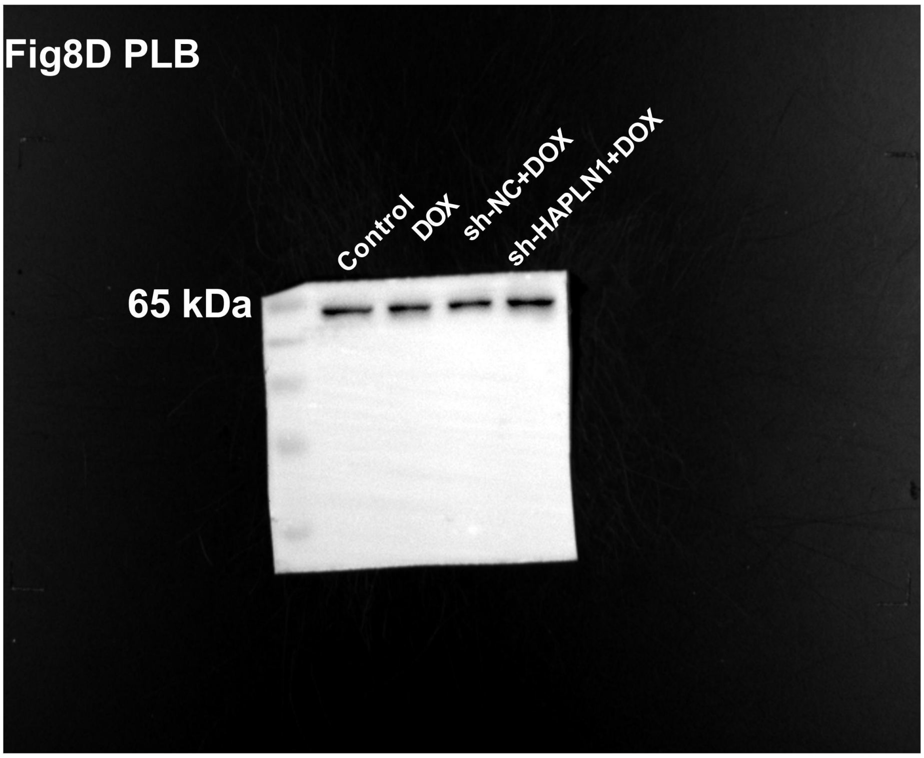

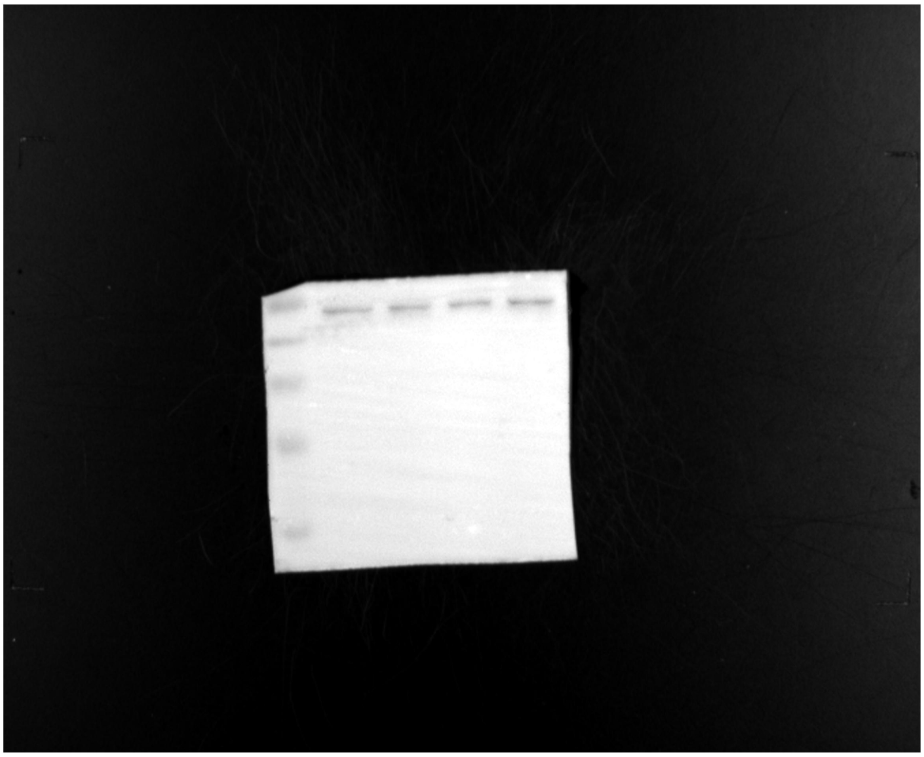

Fig8DCAMK2-include multiple exposures -

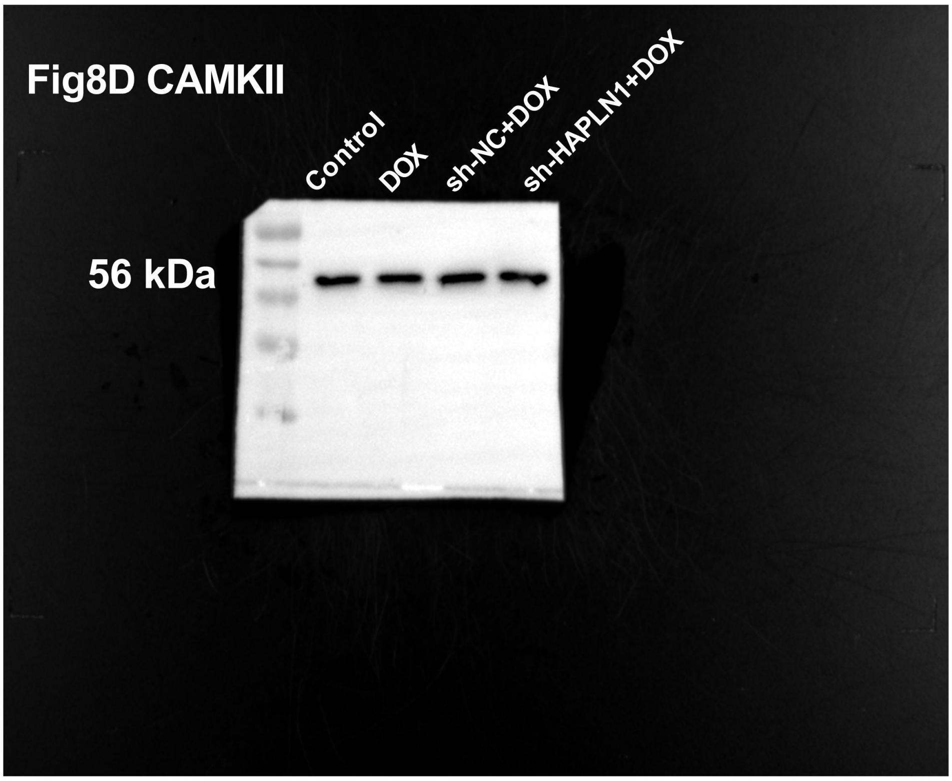

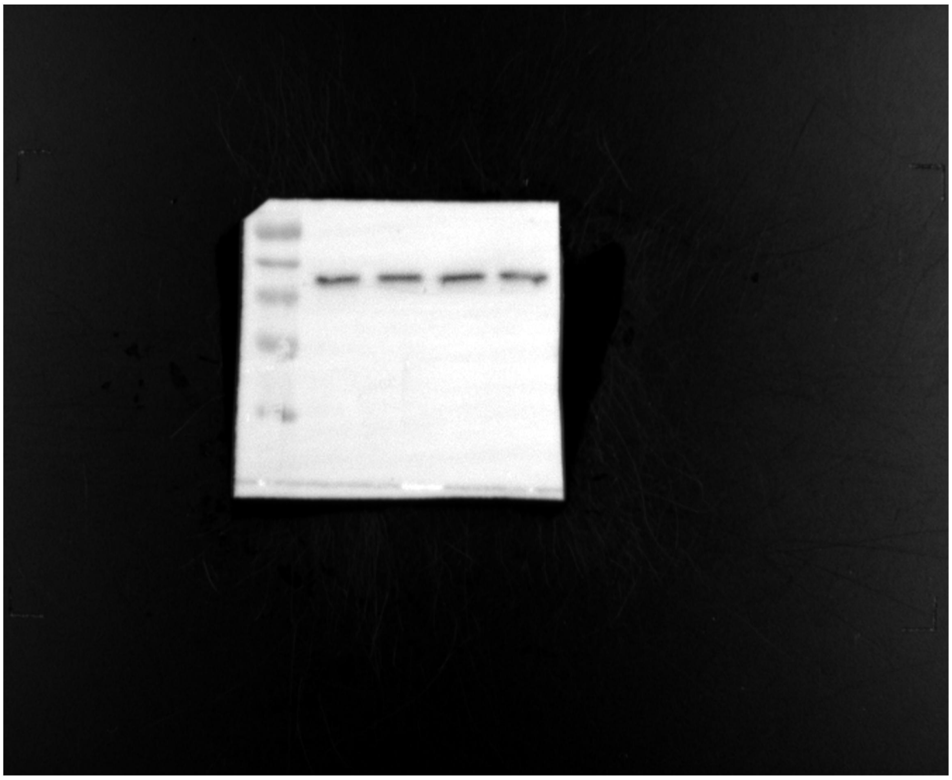

Fig8D p-CAMK-include multiple exposures

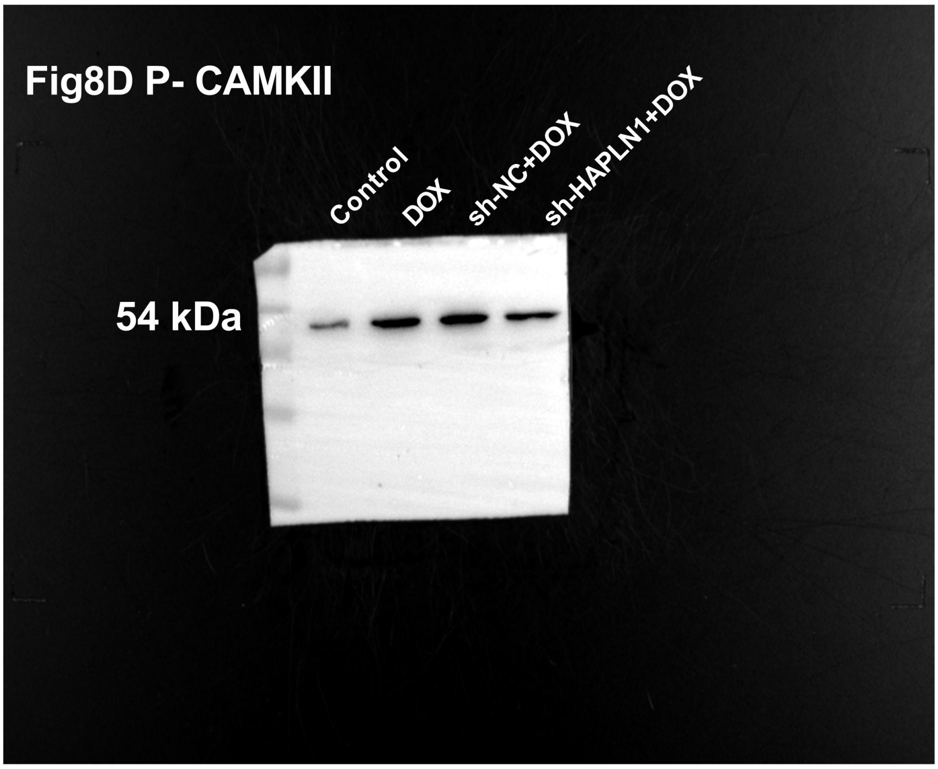

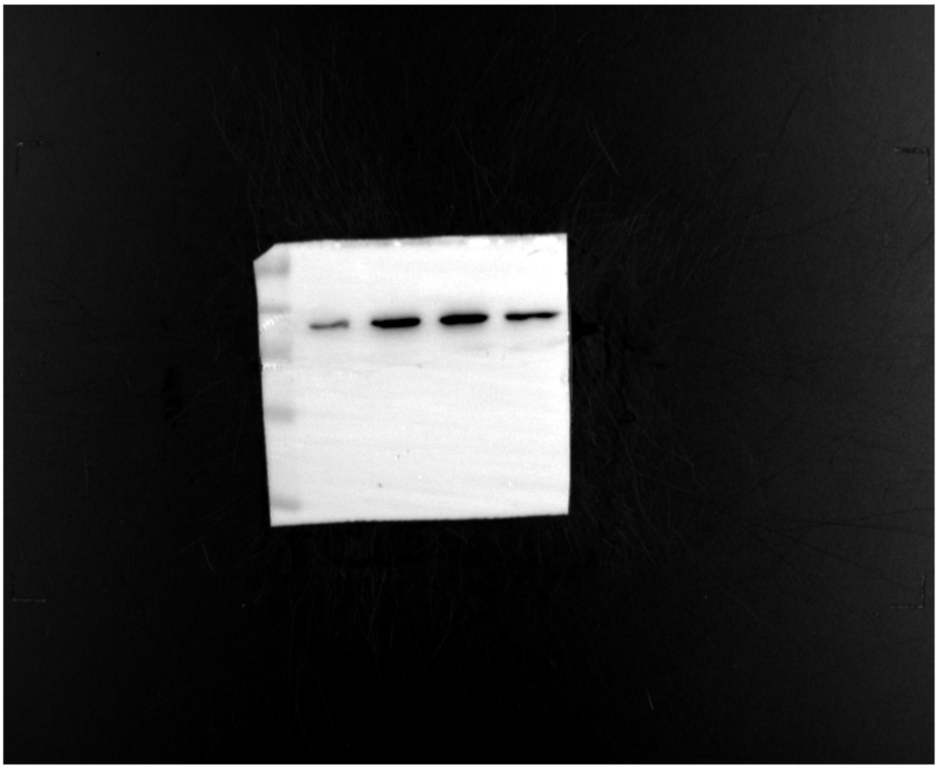

Supplement: Supplementary file 3 — Supplementary Material 3 [file 12872_2024_3861_MOESM3_ESM.pdf]
