# Supplementary material for: HAPLN1 knockdown inhibits heart failure development via activating the PKA signaling pathway
Source: BMC Cardiovasc Disord. 2024 Apr 5;24:197. doi: 10.1186/s12872-024-03861-8 (PMC10996236; doi:10.1186/s12872-024-03861-8)
Supplement: Supplementary file 4 — Supplementary Material 4 [file 12872_2024_3861_MOESM4_ESM.docx]

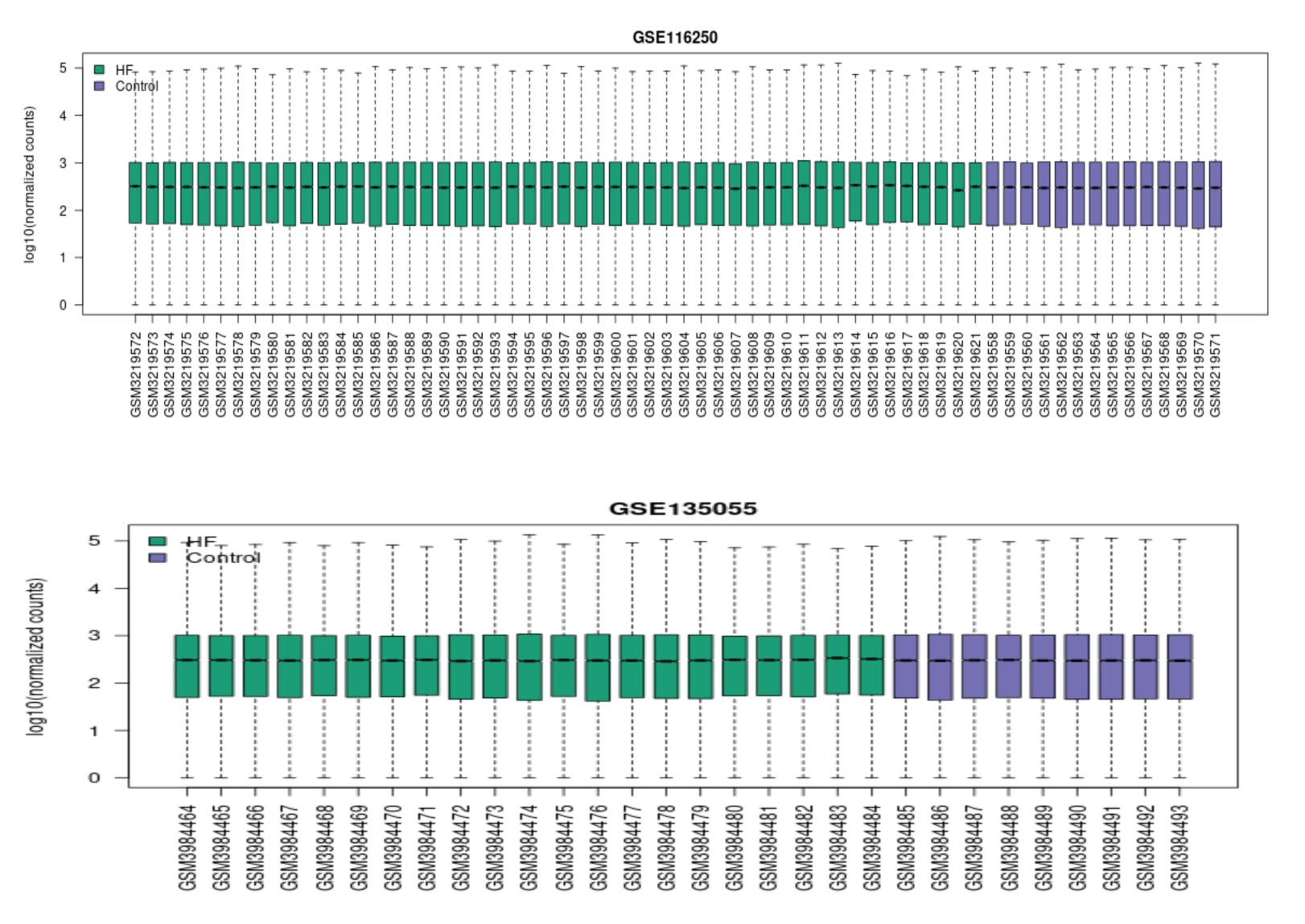


**Supplementary figure 1** Sample data from datasets GSE116250 and GSE135055 were normalized. Each bioinformatics analysis was performed independently three times.


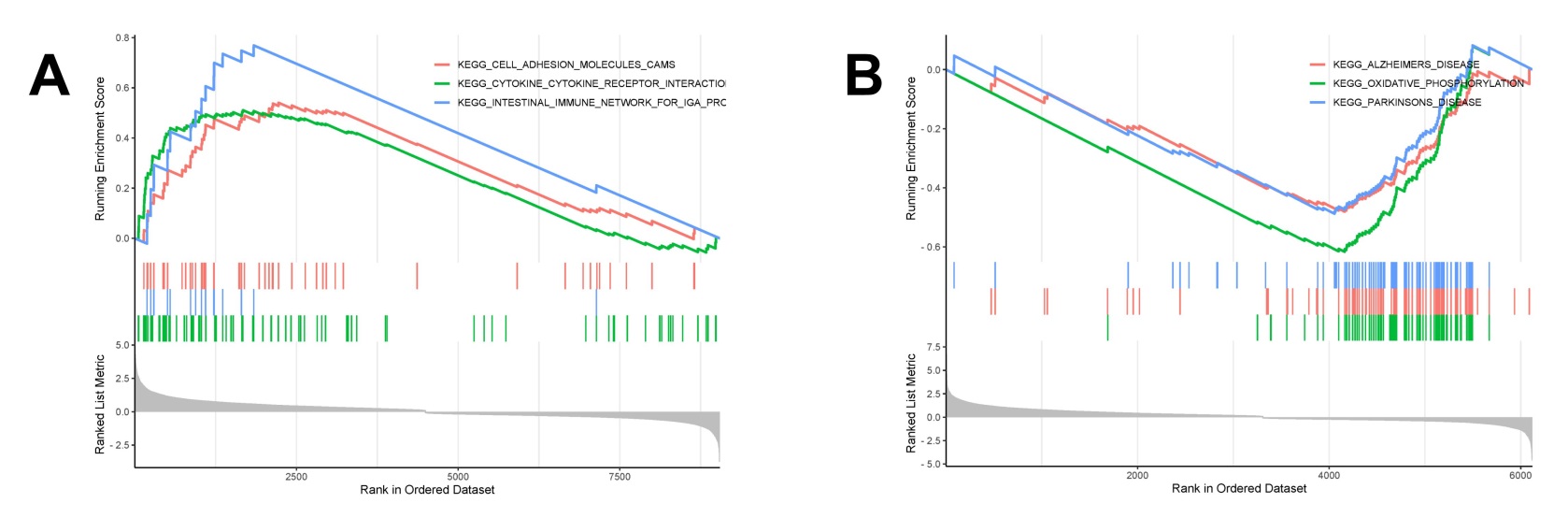


**Supplementary figure 2** GSEA function investigation. A. GSEA analysis of the GSE116250 dataset. B. GSEA analysis of the GSE135055 dataset. Each bioinformatics analysis was performed independently three times. Notes: GSEA analysis, Gene set enrichment analysis.


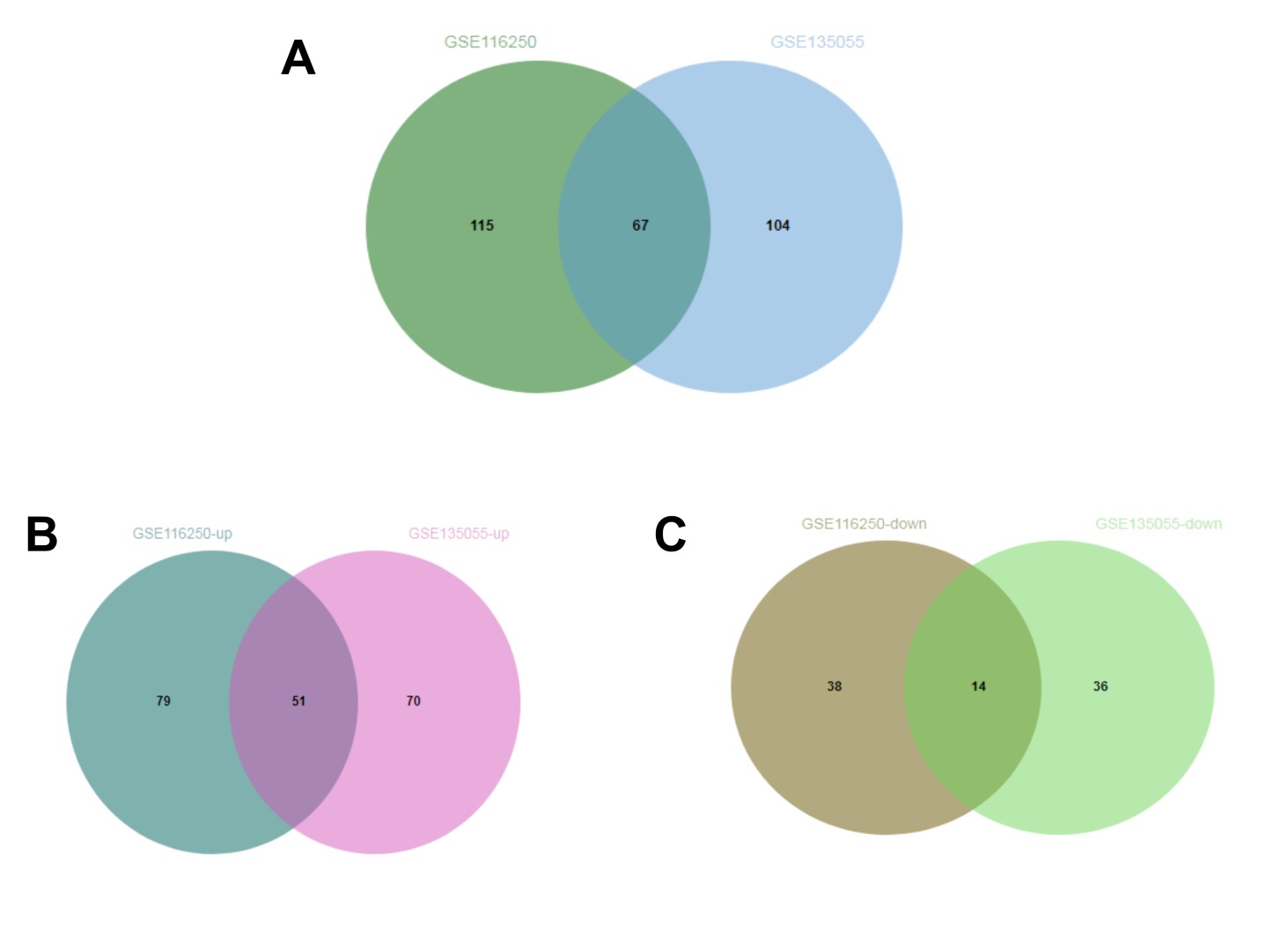


**Supplementary figure 3** DEGs common to the GSE116250 and GSE135055 datasets. A. Total common DEGs. B. The up-regulated DEGs in the two datasets. C. The down-regulated DEGs in the two datasets. Each bioinformatics analysis was performed independently three times. Notes: DEGs, differently expressed genes.


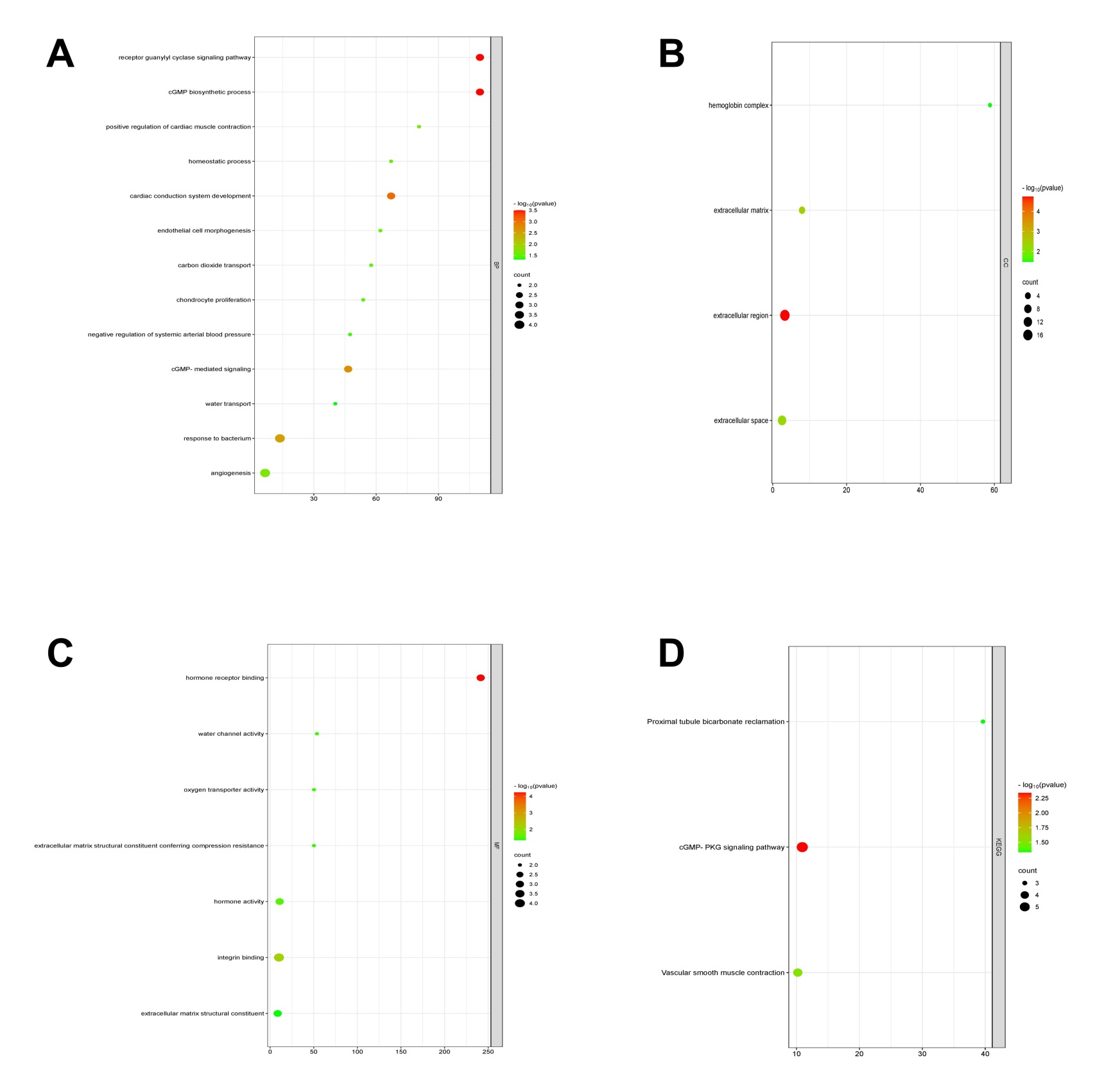


**Supplementary figure 4** GO enrichment and KEGG enrichment analyses of DEGs. A-C. GO-BP, GO-CC, and GO-MF function annotations of the DEGs. D. The KEGG analyses of DEGs. The dot's size and color correspond to the enriched gene count and statistical significance of each term, respectively. Each bioinformatics analysis was performed independently three times. Notes: GO, Gene Ontology; KEGG, Kyoto Encyclopedia of Genes and Genomes; BP, biological process; CC, cellular component; MF, molecular function.


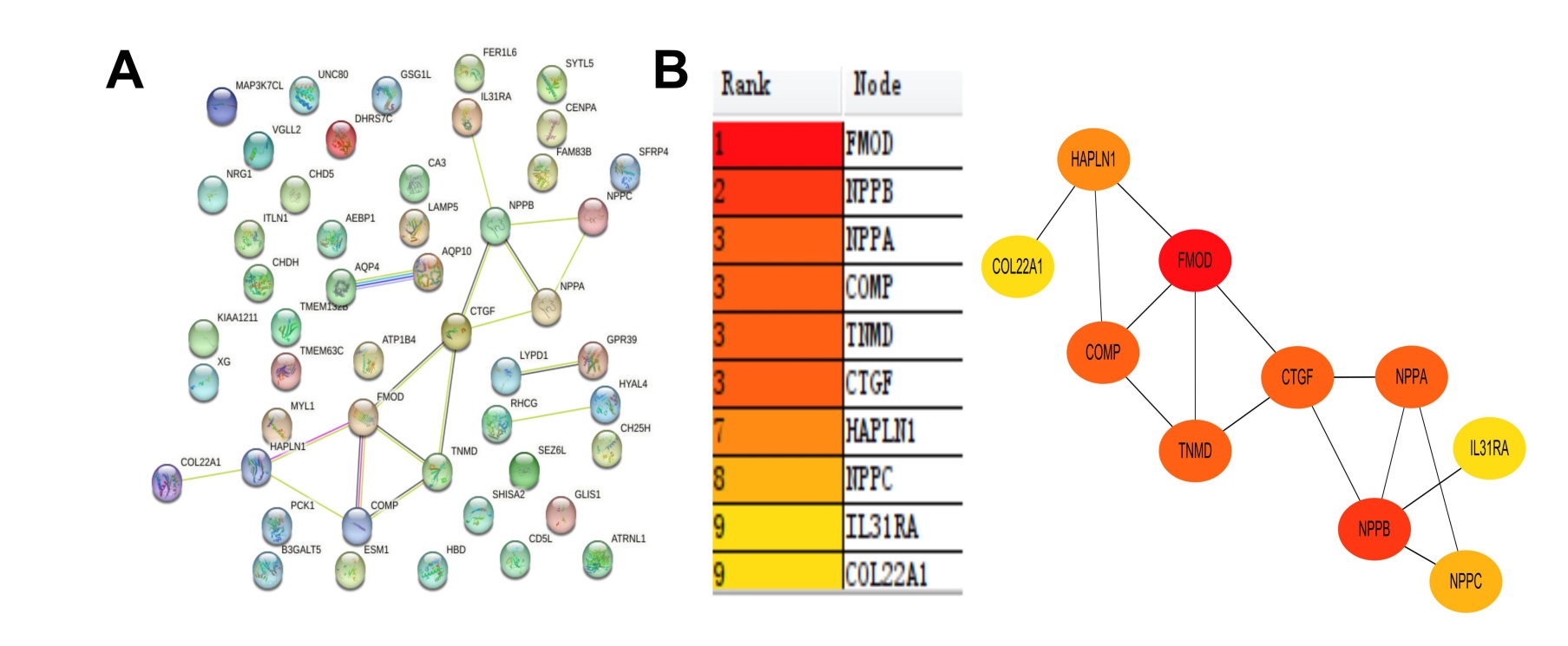


**Supplementary figure 5** Hub genes were screened by constructing a PPI network. A. PPI network of the DEGs. B. Hub genes which were identified based on MCC analysis in CytoHubba plug-in. Each bioinformatics analysis was performed independently three times. Notes: PPI network, protein-protein interaction network; MCC, Maximal Clique Centrality.

Supplementary Table 1 Sequence information of primers used in this study

| Name | Sequences (5’-3’) |
| --- | --- |
| GAPDH-F | GAGTCAACGGATTTGGTCGT |
| GAPDH-R | TTGATTTTGGAGGGATCTCG |
| NPPC -F | CCCTGCTGCTCACGCTGCTCTCCC |
| NPPC -R | CTTCTTGTTGGCTCCTTTGTATTT |
| HAPLN1-F | TCTGAAGGGAGGCAGTGATAGTGA |
| HAPLN1-R | GCGTCGTACAGCTGGTCGAAGGAG |
| FMOD -F | CCTACACCTACGGCTCTCCATCCC |
| FMOD -R | AAGACCTTCCTGCCCACCTTATCA |
| NPPB-F | CCTGCTCTTCTTGCATCTGGCTTT |
| NPPB-F | CACCTGTGGGACGGGGGCTCTCCT |
| NPPA-F | GCTTCCTCCTTTTACTGGCATTCC |
| NPPA-R | CCCCCGCTTCTTCATTCGGCTCAC |
| COMP-F | GCTGTGGGTTACACTGCCTTCAAT |
| COMP -R | GGTTCGCCTGCCAATACGTTTGCT |

Supplementary Table 2 Gene sequences used in silencing HAPLN1 and NC

| Primer name | Sequence (5'-3') |
| --- | --- |
| sh-NC-F | GCTGATCATCTTTCAGACAAC |
| sh-NC-R | GTTGTCTGAAAGATGATCAGC |
| sh-HAPLN1-F-1 | GGATCATGACAGAGCTATTCA |
| sh-HAPLN1-R-1 | TGAATAGCTCTGTCATGATCC |
| sh-HAPLN1-F-2 | GGCAATGTTACACTGCCATGT |
| sh-HAPLN1-R-2 | ACATGGCAGTGTAACATTGCC |
| sh-HAPLN1-F-3 | GGACTTACAAGGTGTGGTATT |
| sh-HAPLN1-R-3 | AATACCACACCTTGTAAGTCC |

Supplementary Table 3 Top 30 DEGs of the GSE116250 dataset

| **Name** | **Description** | **log2FoldChange** | **pval** | **up/down** |
| --- | --- | --- | --- | --- |
| NPPA | natriuretic peptide A | 4.82 | 2.53E-14 | up |
| TNMD | tenomodulin | 4.71 | 3.30E-14 | up |
| HBD | hemoglobin subunit delta | 4.32 | 1.61E-11 | up |
| SEZ6L | seizure related 6 homolog like | 4.25 | 6.90E-26 | up |
| UNC80 | unc-80 homolog, NALCN channel complex subunit | 4.11 | 6.02E-19 | up |
| NPPA-AS1 | NPPA antisense RNA 1 | 4.06 | 8.32E-13 | up |
| TNNI1 | troponin I1, slow skeletal type | 4.00 | 8.83E-15 | up |
| NPPB | natriuretic peptide B | 3.75 | 4.02E-11 | up |
| SFRP4 | secreted frizzled related protein 4 | 3.72 | 5.60E-17 | up |
| COL22A1 | collagen type XXII alpha 1 chain | 3.67 | 3.09E-11 | up |
| AQP10 | aquaporin 10 | 3.57 | 2.48E-16 | up |
| FHAD1-AS1 | FHAD1 antisense RNA 1 | 3.51 | 1.36E-18 | up |
| GSG1L | GSG1 like | 3.49 | 7.50E-09 | up |
| IL31RA | interleukin 31 receptor A | 3.44 | 8.06E-12 | up |
| SHISA2 | shisa family member 2 | 3.33 | 4.23E-13 | up |
| KCNA7 | potassium voltage-gated channel subfamily A member 7 | -2.45 | 7.31E-11 | down |
| SERPINA3 | serpin family A member 3 | -2.45 | 2.26E-08 | down |
| GNMT | glycine N-methyltransferase | -2.52 | 5.23E-18 | down |
| LINC02177 | long intergenic non-protein coding RNA 2177 | -2.57 | 1.05E-08 | down |
| AQP4 | aquaporin 4 | -2.63 | 6.19E-07 | down |
| THRSP | thyroid hormone responsive | -2.69 | 2.75E-05 | down |
| RGR | retinal G protein coupled receptor | -2.71 | 8.06E-12 | down |
| FER1L6 | fer-1 like family member 6 | -2.74 | 3.70E-10 | down |
| CFTR | CF transmembrane conductance regulator | -2.97 | 1.91E-07 | down |
| FGF10 | fibroblast growth factor 10 | -2.98 | 4.25E-10 | down |
| STAC2 | SH3 and cysteine rich domain 2 | -2.98 | 5.14E-15 | down |
| RNASE2 | ribonuclease A family member 2 | -3.11 | 2.36E-11 | down |
| OVOS2 | alpha-2-macroglobulin like 1 pseudogene | -3.43 | 4.72E-12 | down |
| FAM83B | family with sequence similarity 83 member B | -3.43 | 4.88E-12 | down |
| SAA1 | serum amyloid A1 | -3.75 | 1.31E-07 | down |

Supplementary Table 4 Top 30 DEGs of the GSE135055 dataset

| **Name** | **Description** | **log2FoldChange** | **pval** | **up/down** |
| --- | --- | --- | --- | --- |
| NPPB | natriuretic peptide B | 7.55 | 4.84E-38 | up |
| NPPA | natriuretic peptide A | 7.46 | 7.83E-25 | up |
| NPPA-AS1 | NPPA antisense RNA 1 | 6.71 | 2.05E-23 | up |
| CST2 | cystatin SA | 5.06 | 1.07E-06 | up |
| ATP1B4 | ATPase Na+/K+ transporting family member beta 4 | 4.56 | 1.03E-13 | up |
| HBD | hemoglobin subunit delta | 4.31 | 5.12E-06 | up |
| SCG2 | secretogranin II | 4.01 | 6.07E-06 | up |
| CA3 | carbonic anhydrase 3 | 3.88 | 3.68E-08 | up |
| SYTL5 | synaptotagmin like 5 | 3.82 | 2.37E-10 | up |
| MIR5690 | microRNA 5690 | 3.70 | 2.96E-12 | up |
| TNMD | tenomodulin | 3.32 | 3.67E-04 | up |
| COMP | cartilage oligomeric matrix protein | 3.20 | 1.22E-03 | up |
| COL22A1 | collagen type XXII alpha 1 chain | 3.15 | 5.36E-06 | up |
| GSG1L | GSG1 like | 3.10 | 8.80E-04 | up |
| SEZ6L | seizure related 6 homolog like | 3.09 | 3.41E-10 | up |
| SERTM1 | serine rich and transmembrane domain containing 1 | -2.49 | 3.17E-07 | down |
| NR4A2 | nuclear receptor subfamily 4 group A member 2 | -2.49 | 1.36E-10 | down |
| ADAM11 | ADAM metallopeptidase domain 11 | -2.60 | 3.19E-10 | down |
| DHRS7C | dehydrogenase/reductase 7C | -2.61 | 2.08E-03 | down |
| BMP7 | bone morphogenetic protein 7 | -2.67 | 1.16E-06 | down |
| RET | ret proto-oncogene | -2.70 | 1.55E-08 | down |
| LSAMP | limbic system associated membrane protein | -2.74 | 4.32E-09 | down |
| CHST9 | carbohydrate sulfotransferase 9 | -2.84 | 8.28E-09 | down |
| SLC36A2 | solute carrier family 36 member 2 | -2.90 | 8.48E-13 | down |
| ETNPPL | ethanolamine-phosphate phospho-lyase | -3.00 | 1.27E-23 | down |
| TCF24 | transcription factor 24 | -3.33 | 1.42E-12 | down |
| FAM83B | family with sequence similarity 83 member B | -3.78 | 7.33E-12 | down |
| FER1L6 | fer-1 like family member 6 | -4.19 | 4.98E-25 | down |
| CYP1A1 | cytochrome P450 family 1 subfamily A member 1 | -4.43 | 3.34E-04 | down |
| AQP4 | aquaporin 4 | -4.64 | 3.55E-09 | down |

Supplementary Table 5 Hub genes were identified in the cytoHubba plugin of the Cytoscape software

| Gene | MCC | DMNC | MNC | Degree | EPC | BottleNeck | EcCentricity | Closeness | Radiality | Betweenness | Stress | ClusterinfCoefficienet |
| --- | --- | --- | --- | --- | --- | --- | --- | --- | --- | --- | --- | --- |
| FMOD | 6 | 0.2842 | 4 | 4 | 4.646 | 4 | 0.20833 | 6.16667 | 3.05556 | 27 | 40 | 0.5 |
| NPPB | 5 | 0.30898 | 3 | 4 | 4.318 | 2 | 0.15625 | 5.91667 | 2.91667 | 22 | 32 | 0.33333 |
| CTGF | 4 | 0.30779 | 2 | 4 | 4.757 | 10 | 0.20833 | 6.33333 | 3.125 | 40 | 60 | 0.33333 |
| NPPA | 4 | 0.30898 | 3 | 3 | 4.195 | 2 | 0.15625 | 5.41667 | 2.84722 | 6 | 14 | 0.66667 |
| TNMD | 4 | 0.30898 | 3 | 3 | 4.441 | 1 | 0.20833 | 5.5 | 2.91667 | 5 | 12 | 0.66667 |
| COMP | 4 | 0.30898 | 3 | 3 | 4.247 | 1 | 0.15625 | 5.16667 | 2.70833 | 2 | 4 | 0.66667 |
| HAPLN1 | 3 | 0.30779 | 2 | 3 | 3.902 | 2 | 0.15625 | 5.16667 | 2.70833 | 16 | 20 | 0.33333 |
| NPPC | 2 | 0.30779 | 2 | 2 | 3.479 | 1 | 0.125 | 4.36667 | 2.43056 | 0 | 0 | 1 |
| IL31RA | 1 | 0 | 1 | 1 | 2.659 | 1 | 0.125 | 3.86667 | 2.36111 | 0 | 0 | 0 |
| RHCG | 1 | 0 | 1 | 1 | 1.427 | 1 | 0.125 | 1 | 0.375 | 0 | 0 | 0 |
| HYAL4 | 1 | 0 | 1 | 1 | 1.427 | 1 | 0.125 | 1 | 0.375 | 0 | 0 | 0 |
| LYPD1 | 1 | 0 | 1 | 1 | 1.432 | 1 | 0.125 | 1 | 0.375 | 0 | 0 | 0 |
| GPR39 | 1 | 0 | 1 | 1 | 1.432 | 1 | 0.125 | 1 | 0.375 | 0 | 0 | 0 |
| COL22A1 | 1 | 0 | 1 | 1 | 2.532 | 1 | 0.125 | 3.56667 | 2.15278 | 0 | 0 | 0 |
| AQP4 | 1 | 0 | 1 | 1 | 1.435 | 1 | 0.125 | 1 | 0.375 | 0 | 0 | 0 |
| AQP10 | 1 | 0 | 1 | 1 | 1.435 | 1 | 0.125 | 1 | 0.375 | 0 | 0 | 0 |
